# The ATP-mediated formation of the YgjD–YeaZ–YjeE complex is required for the biosynthesis of tRNA t^6^A in *Escherichia coli*

**DOI:** 10.1093/nar/gku1397

**Published:** 2015-01-10

**Authors:** Wenhua Zhang, Bruno Collinet, Ludovic Perrochia, Dominique Durand, Herman van Tilbeurgh

**Affiliations:** 1 Institut de Biochimie et Biophysique Moléculaire et Cellulaire, UMR 8619, CNRS, Bâtiment 430, Université de Paris-Sud, 91405 Orsay Cedex, France; 2 Sorbonne Universités, UPMC Univ Paris 06, UFR 927, Sciences de la vie, F-75005 Paris, France; 3 Institut de Génétique et de Microbiologie, Université Paris-Sud, UMR8621-CNRS, 91405 Orsay, France

## Abstract

The essential and universal *N*^6^-threonylcarbamoyladenosine (t^6^A) modification at position 37 of ANN-decoding tRNAs plays a pivotal role in translational fidelity through enhancement of the cognate codon recognition and stabilization of the codon–anticodon interaction. In *Escherichia coli*, the YgjD (TsaD), YeaZ (TsaB), YjeE (TsaE) and YrdC (TsaC) proteins are necessary and sufficient for the *in vitro* biosynthesis of t^6^A, using tRNA, ATP, *L*-threonine and bicarbonate as substrates. YrdC synthesizes the short-lived *L*-threonylcarbamoyladenylate (TCA), and YgjD, YeaZ and YjeE cooperate to transfer the *L*-threonylcarbamoyl-moiety from TCA onto adenosine at position 37 of substrate tRNA. We determined the crystal structure of the heterodimer YgjD–YeaZ at 2.3 Å, revealing the presence of an unexpected molecule of ADP bound at an atypical site situated at the YgjD–YeaZ interface. We further showed that the ATPase activity of YjeE is strongly activated by the YgjD–YeaZ heterodimer. We established by binding experiments and SAXS data analysis that YgjD–YeaZ and YjeE form a compact ternary complex only in presence of ATP. The formation of the ternary YgjD–YeaZ–YjeE complex is required for the *in vitro* biosynthesis of t^6^A but not its ATPase activity.

## INTRODUCTION

During maturation tRNAs undergo many post-transcriptional modifications, some of which are essential for cell life (1,2). The *N^6^*-threonylcarbamoyladenosine (t^6^A) modification at position 37 of ANN-decoding tRNAs is one of the few modifications that are found in the three domains of life (3–5). The t^6^A base in *Escherichia coli* tRNA^Lys^ stacks with its adjacent A_38_ and forms a cross-strand stack with the first codon of the mRNA, contributing to the translational fidelity (6,7). Furthermore, several isopentenyl-adenosine derivatives at position 37, such as *N^6^*-methyl-*N^6^*-threonylcarbamoyladenosine (m^6^t^6^A) and cyclic t^6^A (ct^6^A), are derivatives of t^6^A in some tRNAs (8,9). Absent or defective t^6^A has been extensively implicated in compromised anticodon–codon interaction, erroneous selection of start codons and aberrant frameshift as well as numerous pleiotropic phenotypes (3,10). It has been shown that the biosynthesis of t^6^A proceeds in two main steps: in the first, members of the Sua5/YrdC protein family, present in bacteria, eukaryotes and archaea, utilize *L*-threonine, bicarbonate and adenosine triphosphate (ATP) to synthesize an unstable intermediate threonylcarbamoyladenylate (TCA) (11,12); in the second step the threonylcarbamoyl moiety of TCA is transferred onto A_37_ of substrate tRNA (11–13). In bacteria, the transfer involves three proteins: YgjD (TsaD), YeaZ (TsaB) and YjeE (TsaE) from *E. coli* (the *Bacillus subtilis* orthologs are YdiE, YdiC and YdiB, respectively) (11,14). In archaea and yeast, the transfer reaction requires the kinase, putative endopeptidase and other proteins of small size (KEOPS) protein complex, composed of Kae1, Bud32, Cgi121 and Pcc1, complemented by a fifth fungi-specific protein Gon7 in yeast (13,15–17). It is now well established that YgjD and its orthologs Kae1 and Qri7 are responsible for the catalysis of the transfer reaction. The contribution of the other protein partners is indispensable but their biochemical function is poorly understood (12–14,18). For example, the mutation of catalytic residues of Bud32, an atypical P-loop kinase (19,20), abolished the formation of tRNA t^6^A *in vitro* and resulted in severe cell growth (13,21), but the need of a kinase activity remains for the moment unexplained. Interestingly, the yeast mitochondrial YgjD/Kae1 paralog, Qri7, is capable of the *in vitro* biosynthesis of tRNA t^6^A on its own if provided with TCA (12,22). The active site of YgjD/Kae1/Qri7 is characterized by a conserved metal-cluster that is contacting the phosphate moieties of a bound ATP (23–25). Mutations of this metal-binding motif abolish the biosynthesis of t^6^A (12,13,18). Mutations or deletions of KEOPS subunits that affect the biosynthesis of tRNA t^6^A have wide ranging biological effects in yeast such as telomere stability, transcription and genomic stability (12,15,16,21,26). It has not been established yet whether KEOPS is directly involved in these processes or whether the phenotypical effects of the deletion mutants are due to impaired t^6^A synthesis.

The essential *ygjD (tsaD), yeaZ (tsaB)* and *yjeE (tsaE)* genes are present in all bacterial genomes, exception made for a few organisms with highly reduced genomes such as *Candidatus Carsonella ruddii* and *Sundamys muelleri* (27–29). The YeaZ protein (231 residues) is a truncated paralog of YgjD (337 residues), having no close orthologs in eukaryotes. A direct interaction between YgjD and YeaZ from *E. coli* has been documented and the crystal structure of heterodimer YgjD–YeaZ from *Salmonella typhimurium* was determined (25,30). The structural comparison of the YgjD–YeaZ heterodimer and the YeaZ homodimer revealed that the same regions of YgjD and YeaZ are involved in dimer formation (25,31). Furthermore, the interacting interface of YgjD–YeaZ is structurally similar to those of the Kae1–Pcc1 complex and of the Qri7 homodimer (12). Bacterial two hybrid studies suggested that YeaZ is capable of interacting with either YjeE or YgjD, forming an essential network that is regulated by ATP turnover by YjeE (32,33). YjeE is only present in bacteria and was reported to possess an intrinsically weak ATPase activity (28,33,34). The crystal structure of YjeE from *Haemophilus* *influenzae* in complex with Adenosinediphosphate (ADP) reveals the presence of a Walker A motif and two switch regions that are the characteristic of P-loop Guanosinetriphosphate hydrolases (GTPases) (35,36). The hydrolysis of ATP into ADP is observed only when YgjD, YeaZ and YjeE are mixed, regardless of the presence of other substrates for the biosynthesis of tRNA t^6^A (14). The contribution of the hydrolysis of ATP to the overall biosynthesis of t^6^A in bacteria is presently unclear.

We report here the crystal structure of the heterodimer YgjD–YeaZ from *E. coli* in complex with ADP that is bound at the interfacial region of YeaZ. We characterized the activation and regulation of the ATPase activity of YjeE by the heterodimer YgjD–YeaZ. The nature of this atypical ADP-binding site at the YgjD–YeaZ interface suggests this might also be the binding site for YjeE. We made a 3D model of the YgjD–YeaZ–YjeE complex that is compatible with the experimental Small Angle X-ray Scattering (SAXS) data. Furthermore, we show that the Mg^2+^-binding site in the putative catalytic center of YgjD is essential for the biosynthesis of tRNA t^6^A. Eventually, we demonstrate that the ATP-mediated dynamic formation of the ternary complex YgjD–YeaZ–YjeE is required for tRNA t^6^A modification but not its ATPase activity.

## MATERIALS AND METHODS

### Cloning, mutagenesis and expression

*Escherichia coli* genes *ygjD* (*tsaD*), *yeaZ* (*tsaB*), *yjeE* (*tsaE*) and *yrdC* (*tsaC*) were amplified from *E. coli* K-12 genomic DNA and cloned into vectors pET9a, pET9a, pET28a and pET26b, respectively (17). Shortly, *ygjD, yeaZ* and *yrdC* were subcloned into the corresponding vectors above with an addition of 6xHis tags at the C-terminus whereas *yjeE* was subcloned into its vector with an addition of Strep II tag at the C-terminus. YgjD, YeaZ, YjeE and YrdC were produced at 37°C in *E. coli* BL21(DE3) Competent Cells (Invitrogen) transformed with pET9a–ygjD, pET9a–yeaZ and, pET28a–yjeE and pET26b–yrdC, respectively. The yields are around 1 mg per liter for all the four proteins (See details in the Supplementary Materials).

The following mutations have been inserted using the Quikchange mutagenesis kit (Agilent Technologies): YgjD–F100E; YgjD–S97E; YgjD–S97R; YgjD–E12A; YgjD–V85E; YeaZ–Δ220; YeaZ–R118A; YjeE-E108A; YjeE–T43A; YjeE–Y82A and YjeE–W109A. Primers (sequences are summarized in supporting materials) have been ordered from Eurofins Genomics, Les Ulis, France. Plasmid template (10 ng) has been incubated with 0.04 μM of each forward and reverse primer, 1 μM of dNTP mix (Thermo Scientific) and 1 unit of Phusion High Fidelity DNA polymerase (Thermo Scientific) in HF buffer. Twenty-five cycles of amplification have been carried out, corresponding to 1 min of denaturation at 95°C, 1 min of annealing at 55°C and 2′30″ of extension at 72°C. Polymerase chain reaction (PCR) tubes have been incubated for 5 more minutes at 72°C as a final extension step. Parental plasmid has been digested for 2 h at 37°C with DpnI restriction enzyme (Thermo Scientific) and 5 μl of the PCR mix has been used to transform XL10 chimio-competent cells (Agilent Technologies). For the screening procedure, four to eight independent colonies have been grown in LB medium supplemented with the appropriate antibiotic and plasmid have been extracted using the Genejet plasmid miniprep kit (Thermo Scientific). Plasmids have been sequenced (Beckman Coulter Genomics) on the whole gene to check for the presence of the right mutation. In few cases an alternative protocol has been used where two separate PCRs have been carried out in parallel using only the forward and the reverse primer in two separate tubes and 100 ng of plasmid template. In this case 30 cycles of amplification have been done and the two tubes have been pooled, heated to 95°C and slowly cooled down before DpnI digestion (37). The rest of the protocol was the same as the one mentioned above. In all cases, all the genes have been sequenced on their full length.

The expression and purification details of all proteins are presented in the Supplementary Materials sections.

### Native gel migration assay

A 1.2% agarose gel was prepared in 30 mM Tris–HCl pH 8.0 and 50 mM Glycine. Protein samples supplemented with 25% glycerol were loaded onto the gel. Migration lasted for 50 min at 135 mV in a running buffer composed of 30 mM Tris–HCl pH 8.0 and 50 mM Glycine. For analysis of the ATP/ADP-dependent association of the proteins, both the gel and the loading protein samples were additionally supplemented with 1.0 mM of ATP or ADP and 2.0 mM MgCl_2_. The gel was running in an electrophoresis apparatus on ice with an ambient temperature at 8°C. Proteins were revealed by Coomassie staining.

### Biophysical and structural analysis

Crystallography, SAXS and biophysical measurements (ITC) are presented in the Supplementary Materials sections. The PDB coordinates were deposited to www.PDB.org for the YgjD(ADP)–YeaZ(ADP), YgjD^E12A^(ATP)–YeaZ and YgjD^V85E^(ATP)–YeaZ crystal structures under accession number of 4WOS, 4WQ4 and 4WQ5, respectively.

### ATPase activity assay

The ATPase activity (conversion into ADP and phosphate) was measured using a Nicotinamide adenine dinucleotide dehydrogenase (NADH)-coupled assay (38). The hydrolysis of ATP into ADP was coupled to the oxidation of NADH to NAD^+^ and the consumption of NADH was collected by monitoring the absorbance at A_340_ by a Cary WinUV 200 spectrophotometer (Agilent Technology, Inc.). Assays were performed in a 10mm cuvette at 20°C. The 150 μL reaction buffer contained 50 mM HEPES pH 7.5, and an excess of pyruvate kinase and lactate dehydrogenase (Sigma). The reactions were monitored after the steady-state reaction was achieved and the linear curves of absorbance at A_340_ were recorded for 30 min. The concentration of the proteins in the assays was 1 μM except for YjeE and its variants, which were at 2 μM. The reaction velocity was calculated by converting the decrease in the absorbance to consumption of NADH using an extinction coefficient of ε_340nm_ = 6220 M^−1^·cm^−1^. ATPase reaction rates as a function of ATP concentration was fitted using Michaelis–Menten equation and the cooperactivity of ATPase activity of YjeE by the dimer YgjD–YeaZ was fitted by Allosteric sigmoidal kinetics equation using the Prism program (GraphPad). All the experiments were done in triplicate and control experiments were carried out in which either proteins or chemicals were omitted from the reaction.

### tRNA overexpression and purification

The *E. coli* tRNA^Lys(UUU)^ was subcloned in a pBlue-Script vector harboring T5 promoter/lac operator sequence (17). The pB–tRNA^Lys(UUU)^ was transformed into *E.coli* XL10 competent cells and was grown in 10ml 2xYT medium at 37°C overnight. The 10 ml preculture was transferred to 800 ml Overnight Express Instant TB medium (Novagen) and the overexpression was auto-induced during the culturing for 36 h at 37°C. The cell culture was harvested and centrifuged for 25 min at 4400 rpm. The cell pellets were suspended in Trizol (Sigma), followed by extraction of the total RNAs according to the manufacturer's procedure (TRI Reagent, Sigma). The total RNA pellet was dissolved in water and applied for purification by a denaturing gel that is composed of 10% of polyacrylamide and 8M Urea. After migration for 4 h at 250 V, 25 mA in Tris-acetate-EDTA (TAE) buffer (40 mM Tris, 20 mM acetic acid and 1 mM ethylenediaminetetraacetic acid (EDTA)), the gel slice containing tRNAs as visualized by UV light shadowing at 258 nm and was cut out and incubated with elution buffer (10 mM Tris–HCl pH 7.5, 300 mM NaCl, 1 mM EDTA and 1% sodium dodecyl sulphate (SDS)) overnight at an ambient temperature. The eluted tRNAs were precipitated with absolute ethanol and NaCl. Finally, the tRNAs were further purified by size exclusion chromatography equilibrated with buffer that is composed of 10 mM HEPES pH 7.5, 100 mM NaCl. The size and purity of the tRNA was analyzed by 1.5% agarose gel.

### The tRNA t^6^A assay

The *in vitro* biosynthesis of tRNA t^6^A, the digestion of tRNA and the high performance liquid chromatography/mass spectrometry (HPLC/MS) analysis of the t^6^A formation used published protocols and are presented in the Supplementary Materials sections.

## RESULTS

### YgjD forms a stable heterodimer with YeaZ

**Figure 1.**
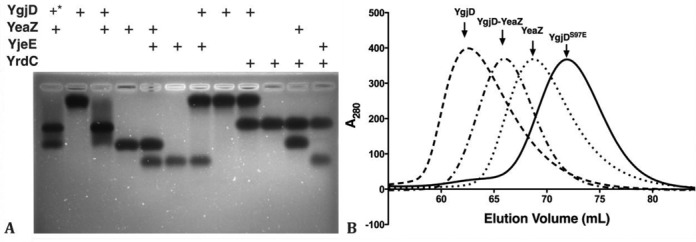
The *in vitro* characterization of protein–protein interactions between YgjD, YeaZ, YjeE and YrdC. (**A**) The native agarose gel shift analysis of various protein mixtures. + signs define the composition of the protein mixtures. The molar ratio in each mixture was 1:1, except *, which was one-half for YgjD. (**B**) Superposed HiLoad 16/60 Superdex 75 pg size-exclusion chromatographic profiles of YgjD, YeaZ, YgjD–YeaZ and YgjD^S97E^.

The four *E. coli* proteins YrdC, YgjD, YeaZ and YjeE are necessary and sufficient for the *in vitro* synthesis of tRNA t^6^A (14). YgjD and YeaZ are forming a heterodimer that is capable of interacting with YjeE (33). In order to get insight into the biochemical function of YrdC, YgjD, YeaZ and YjeE we decided to investigate their interactions *in vitro*. We first expressed and purified the four recombinant proteins. The C-terminal *6xHis*-tagged YgjD is stable and is not sensitive to proteolysis as reported previously (33). We tested the capability of various combinations of the four proteins to form complexes by native gel shift experiments. A mobility shift is observed upon mixing YgjD and YeaZ, demonstrating they are spontaneously forming a complex (Figure 1A, lanes 1–4). No other combination of proteins provoked shifts in the migration.

In order to further characterize the complex formed by YgjD and YeaZ, we subjected it to size-exclusion chromatography. These data confirm that YgjD and YeaZ alone are homodimers and that a heterodimer readily forms upon equimolar mixing of YgjD and YeaZ (Figure 1B). The interaction between YgjD and YeaZ was quantified by ITC (Table 1 and Supplementary Figure S1). Despite the fact that both YgjD and YeaZ form homodimers the titration of YgjD with YeaZ in absence of nucleotides (ADP or ATP) resulted in a binding curve that could be fitted by a simple equilibrium scheme (YgjD–YeaZ complex with 1:1 stoichiometry and a Kd of 122 nM). These binding parameters are similar as those measured for the interaction between YgjD and YeaZ from *S. typhimurium* (Kd of 300 nM) (25).

**Table 1.** The thermodynamic parameters for the protein–protein interaction measured by ITC

Titrant (uM)	Injectant (uM)	Stoichiometry (n)	Kd (uM)	Δ*H* (kcal/mol)	Δ*S* (kcal/mol/deg)
YgjD (30)	YeaZ (360)	0.91 ± 0.0051	0.12 ± 0.03	6.28 ± 0.10	15.1
YgjD (20)	YeaZ^1–219^ (200)	1.06 ± 0.0064	0.03 ± 0.01	5.96 ± 0.12	14.2
YgjD–YeaZ (23)^a^	YjeE (270)^a^	1.01 ± 0.015	0.60 ± 0.034	10.16 ± 0.212	62.6
YgjD–YeaZ (32)^a^	YjeE^W109A^ (380)^a^	0.99 ± 0.015	0.20 ± 0.016	6.47 ± 0.134	47.8
YgjD–YeaZ^R118A^ (40)^a^	YjeE (480)^a^	0.96 ± 0.010	0.93 ± 0.099	11.35 ± 0.227	65.7

The corresponding thermograms are presented in Supplementary Figure S1 for the upper panel and Supplementary Figure S5 for the lower panel.

^a^in presence of 1.0 mM AMPPNP and MgCl_2_.

### Crystal structure of the heterodimer YgjD–YeaZ in complex of ADP

**Figure 2.**
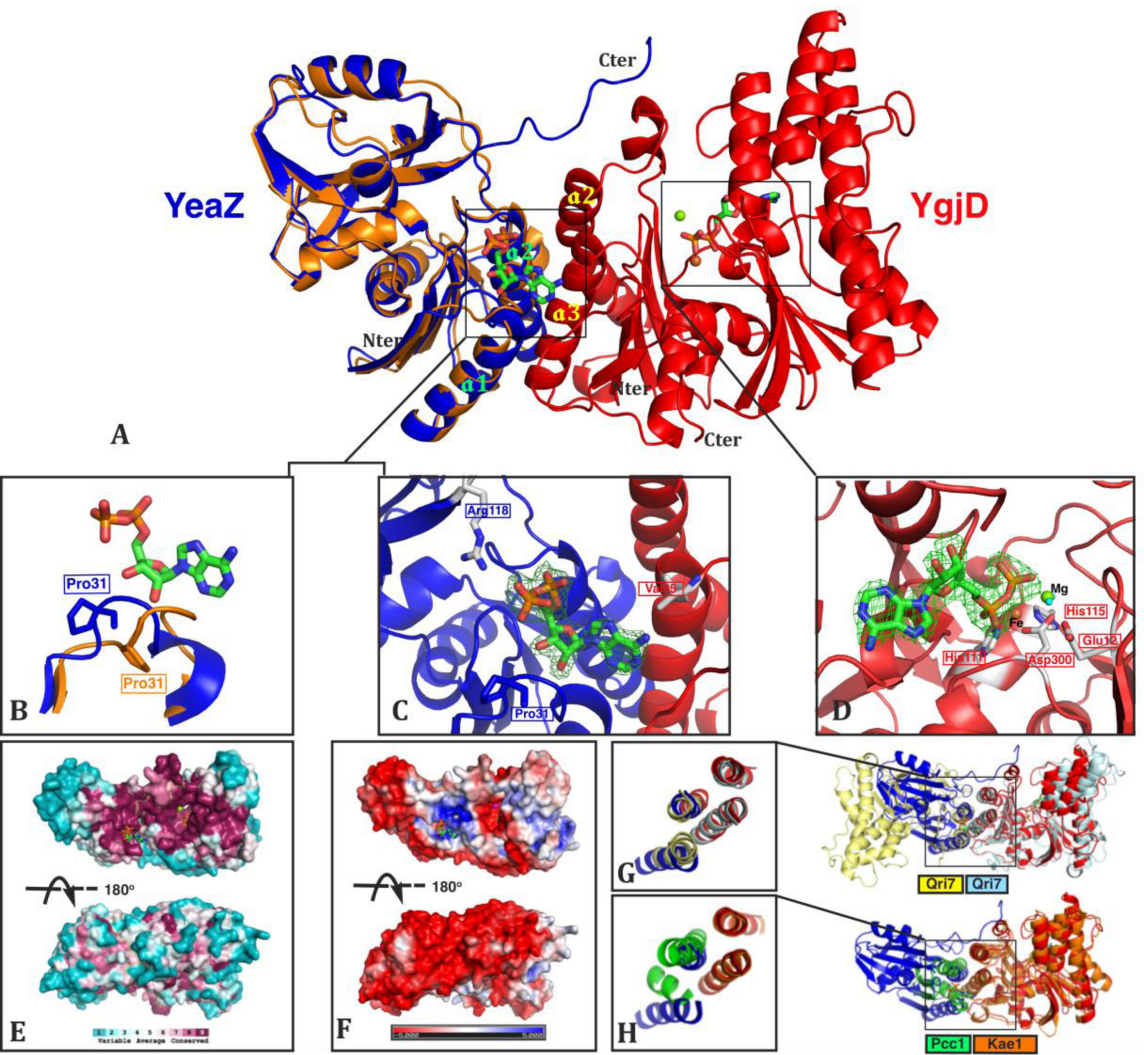
The crystal structure of the heterodimer YgjD–YeaZ in complex with two molecules of ADP. (**A**) Structural superposition of the YeaZ monomer as observed in YeaZ homodimer (gold) onto the YgjD–YeaZ heterodimer (red and blue). (**B**) Conformational change of the loop ^29^LCPREHT^35^ (linking β3 and α1) of YeaZ upon its dimerization with YgjD. (**C** and **D**) *Fo–Fc* difference maps contoured at 3.0 σ for ADP molecules bound at the YgjD–YeaZ interface (C) and the active site of YgjD (D). Residues important for the interaction are represented in sticks. (**E**) The sequence conservation projected onto the surface of the YgjD–YeaZ heterodimer. The conservation is scaled according to the key at the bottom and represented by colors with cyan for variable and raspberry for conserved. (**F**) The electrostatic potential surface of the YgjD–YeaZ heterodimer (red negatively and blue positively charged); back and front views are represented, front views (top) are in the same orientation as for views A. (**G** and **H**) Representation of the similar helical bundle interfaces in the YgjD–YeaZ and Qri7 homodimer or the Kae1–Pcc1 complexes.

We cocrystallized the YgjD–YeaZ heterodimer with ATP and MgCl_2_ and determined the structure at a resolution of 2.3 Å. The overall structure of the YgjD–YeaZ heterodimer and the binding modes of nucleotides (ATP, ADP and AMP) are very similar to those of YgjD–YeaZ from *S. typhimurium*, with an rmsd of 0.232 Å for 571 superposed C_α_ atoms (Figure 2A) (25). We will therefore only describe the most important structural differences between the two homologous complexes. The interface between YgjD and YeaZ resembles that of the most commonly observed YeaZ homodimer and of the Qri7 homodimer (Figure 2A and G) (31,39). Interestingly the Kae1 interface region of the Kae1–Pcc1 complex is the same as for YgjD–YeaZ (Figure 2H). Pcc1 is not related to YeaZ, but its binding to Kae1 strongly mimics that of YgjD and YeaZ. This suggests that, comparable to the essentiality of Qri7 homodimerization, YeaZ and Pcc1 could play a similar role through their interaction with YgjD and Kae1, respectively.

### The YgjD/YeaZ interface creates an atypical ADP-binding site

As expected we observed clear electron density for a nucleotide bound to two metal ions in the active site of YgjD, which we interpreted as Mg^2+^ and Fe^2+/3+^ based on electron density levels and X-ray fluorescence scanning at the Fe-edge (data not shown). Fe^2+/3+^ is liganded by the conserved His^111^, His^115^ and Asp^300^ residues. The β-phosphate group of the nucleotide completes the Fe^2+/3+^-coordination sphere (Figure 2D and Supplementary Figure S2B). The Mg^2+^ ion is located 6.2 Å away from the Fe^2+/3+^ ion and is liganded by the carboxylate side chains of Asp^11^ and Glu^12^, and close (3.7 Å) to the β-phosphate oxygen of the nucleotide. Two water molecules are involved in the coordination of the Mg^2+^ ion (Figure 2D and Supplementary Figure S2B). We did not observe electron density for the γ-phosphate group of ATP, which probably has been converted into ADP during the crystallization process. The ADP bound in YgjD is well superposable to the ATP bound in *Pa*–Kae1. The Fe^3+^ ion in *Pa*–Kae1 is coordinated by a tyrosinate bond (24). This tyrosine is conserved in eukaryotic and archaean Kae1 orthologs but neither in bacteria, nor in the Qri7 mitochondrial ortholog.

Interestingly, the *Fo–Fc* difference map calculated after structural refinement revealed an important residual electron density cloud situated at the YgjD–YeaZ interface (Figure 2C). An ADP molecule could unequivocally fit the density and its refined B-factors were similar to those of the surrounding side chains. ADP is bound on top of the YeaZ helical bundle at the heterodimer interface. The ADP nucleotide interacts mainly with the N-terminal domain of YeaZ, but there are also a few interactions with YgjD. The α-phosphate moiety of this ADP is very close to the best conserved sequence motif ^64^GPGS(Y/F)TG(I/L/V)R^72^ of YeaZ, reminiscent of a phosphate-binding motif (40). The β-phosphate is forming an ionic interaction with YeaZ^Arg118^ and the ribose 2′- and 3′-OH groups are forming hydrogen bonds with the YeaZ^His34^ side chain and the main chain carbonyl of YeaZ^Arg32^, both these residues are also very well conserved. The adenine base lies on top of a hydrophobic depression created at the interacting interface of YgjD–YeaZ (YeaZ^Thr69^ and YgjD^Leu89^) but does not form any specific H-bond interactions with the heterodimer. Upon superposing the YgjD–YeaZ heterodimer and the YeaZ homodimer, we noticed that there is a large conformational change in the YeaZ loop connecting α3 and α1 (Figure 2B residues Cys^30^–Thr^3^^5^). While this loop is in an open conformation in the heterodimer YgjD–YeaZ, it adopts a closed conformation in the YeaZ homodimer that overlaps with the interfacial ADP binding site in the heterodimer YgjD–YeaZ (31,41). A similar conformational change was observed for a comparable loop in YeaZ in the *St–*YgjD–YeaZ heterodimer which did not have any nucleotide bound at the interface (25). Instead, *St*–YgjD–YeaZ has a tris(hydroxyethyl)aminoethane molecule bound which partially overlaps with the phosphate groups of ADP at the *Ec*–YgjD–YeaZ dimer interface.

### Thermodynamics of nucleotide binding

In order to better understand the role of ATP in the transfer of the threonylcarbamoyl-moiety from TCA onto tRNA, we characterized the nucleotide-binding properties of the YgjD, YeaZ, YjeE and YgjD–YeaZ by ITC. Thermograms (Supplementary Figure S3) and thermodynamic values (Table 2) were all obtained in presence of 500 μM Mg^2+^. The titration of YgjD or YgjD–YeaZ heterodimer with ATP or ADP did not produce any heat exchange signals (data not shown for ATP and Supplementary Figure S3G for ADP). There was no heat exchange upon mixing of YeaZ with any of the tested nucleotides (Supplementary Figure S3H and S3I). Hence, the binding of ADP to the YgjD–YeaZ interface could not be detected by ITC experiments. However, AMPCPP bound to YgjD with a Kd of 1.6 μM and a Δ*H* of 2.45 Kcal/mole, and to the YgjD–YeaZ heterodimer with a Kd of 0.7 μM and a ΔH of 0.9 Kcal/mole. The heterodimerization with YeaZ does not seem to significantly affect the affinity of YgjD for AMPCPP.

**Table 2.** The thermodynamic parameters for the binding of nucleotides to YgjD, YeaZ, YgjD–YeaZ, YjeE and the mutants measured by ITC

Titrant (uM)	Injectant (uM)	Stoichiometry (n)	Kd (uM)	Δ*H* (kcal/mol)	Δ*S* (kcal/mol/deg)
YgjD (30)^a^	AMPCPP (360)	2.07 ± 0.024	1.62 ± 0.088	2.45 ± 0.045	34.9
YgjD–YeaZ (30)	AMPCPP (360)	1.41 ± 0.062	0.70 ± 0.011	0.91 ± 0.066	31.2
YjeE (57)^a^	ATPγS (680)^a^	1.06 ± 0.027	20.41 ± 1.32	−2.07 ± 0.067	15.4
YjeE (50)^a^	ADP (600)^a^	0.86 ± 0.026	6.54 ± 0.053	−3.94 ± 0.159	10.5
YjeE^Y82A^ (28)^a^	ATPγS (350)^a^	1.05 ± 0.154	8.90 ± 0.013	−1.26 ± 0.297	19.0
YjeE^W109A^ (41)^a^	ADP (492)^a^	0.82 ± 0.038	4.83 ± 0.022	−5.19 ± 0.327	6.93

The corresponding thermograms are presented in Supplementary Figure S3.

^a^in presence of 500 uM MgCl_2_.

YjeE is structurally related to small GTPases (Supplementary Figure S2C and S2E) and exhibits intrinsically weak ATPase activity (34–36). However, the titration of YjeE with Guanosine diphosphate (GDP) or ATP in presence of Mg^2+^ did not produce any heat exchange signals. By contrast, the titration of YjeE with ADP or ATPγS yielded Kds of 6.54 and 20.41 μM and Δ*H* values of −3.94 Kcal/mole and −2.07 Kcal/mole, respectively (Supplementary Figures S3C and S3D and Table 2). This also confirms that YjeE preferentially binds to ADP rather than ATP and it also correlates with the fact that most small G-proteins preferentially bind to GDP compared to GTP (34,42).

### The ATPase activity of YjeE is activated and regulated by the YgjD–YeaZ heterodimer

**Figure 3.**
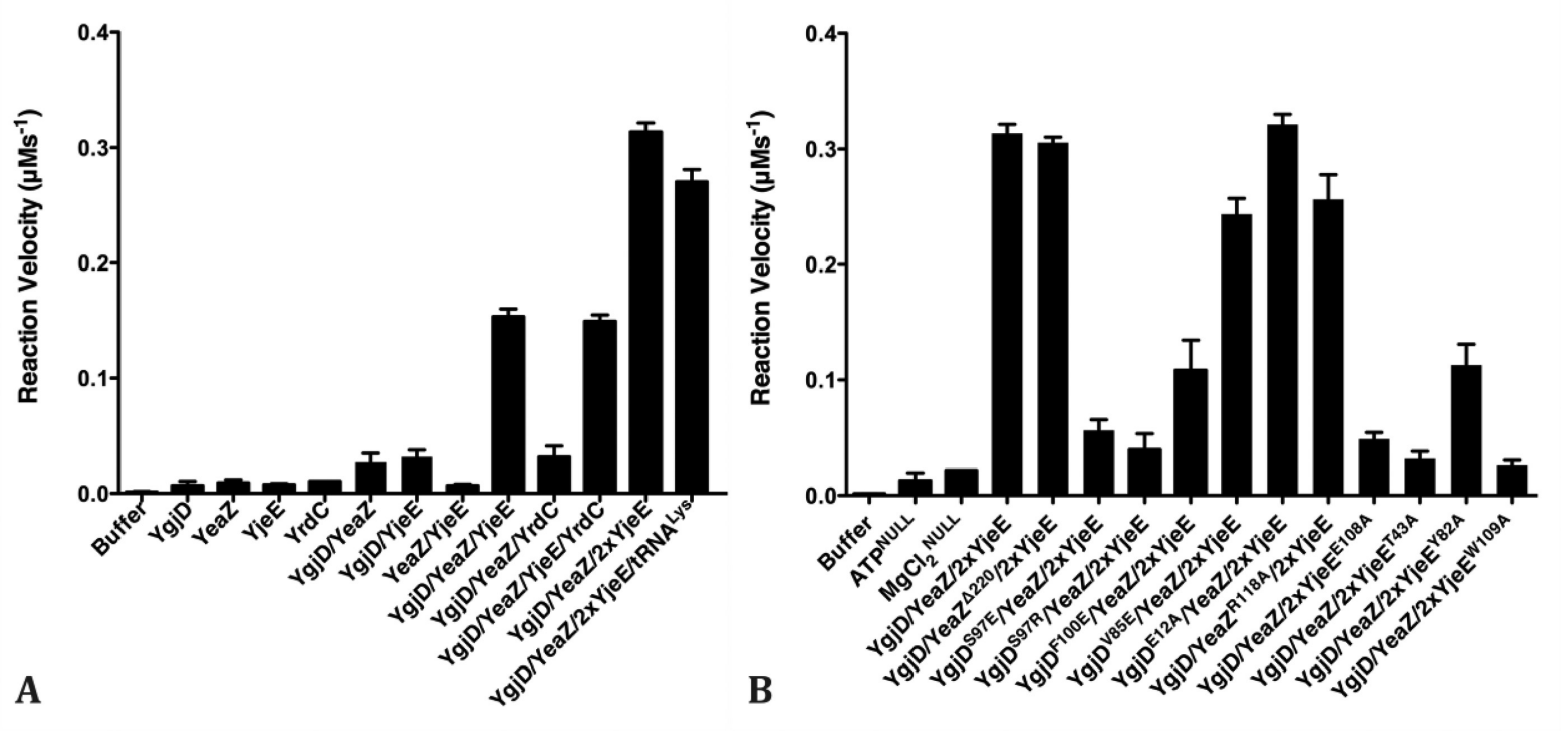
The ATPase activities of YgjD, YeaZ, YjeE and YrdC, and their mutants. (**A**) ATPase activity of different combinations of wild type proteins. 2xYjeE denotes a molar ratio of YjeE of 2. (**B**) ATPase activity of different combinations of mutants. Null denotes absence of a chemical. 2xYjeE was subjected to guarantee a 1:1 molar ratio of YjeE monomer to YgjD/YeaZ mixture. The bars represent the standard deviations from triplicates.

The t^6^A biosynthesis systems require ATPase activities. One ATP is chemically consumed for the synthesis of the TCA intermediate by YrdC/Sua5. A second ATP molecule is hydrolyzed during the threonylcarbamoyl transfer from TCA onto tRNA (13,14). The hydrolysis of the latter ATP molecule is not needed for the chemistry of the transfer reaction and might therefore play a regulatory role (13). To better understand the need for ATPase activity in bacterial biosynthesis of t^6^A, we measured the hydrolysis of ATP into ADP by YgjD, YeaZ, YjeE and YrdC using an NADH-coupled ATPase activity assay. Individual YgjD, YeaZ, YjeE and YrdC do not exhibit ATPase activity whereas YgjD exhibits very weak ATPase activity in presence of YeaZ or YjeE (Figure 3A). However the ATPase activity of YjeE is strongly activated by the YgjD–YeaZ heterodimer and this effect is maximal for a 1:2 YgjD–YeaZ:YjeE stoichiometry. Kinetic characterization of the ATPase activity of the YgjD–YeaZ–2xYjeE complex against ATP concentration yielded a *K*_m_ and *k*_cat_ of 0.644 mM and 0.4 s^−1^ (Supplementary Figure S4), in comparison with reported values of YjeE alone of 1.4 mM and 0.003 s^−1^ respectively (34). The ATPase activity is not affected by the addition of either YrdC or tRNA^Lys^ (Figure 3A). We observed that YjeE exists in an equilibrium between monomer and dimer (Figure 6A and Supplementary Figure S4C). Consistent with this observation the ATPase activity of YjeE is also proportional to the percentage of monomeric YjeE (Supplementary Figure S4C), suggesting monomeric YjeE binds to and is activated by YgjD–YeaZ. Similarly, the monomeric form of the *B. subtilis* ortholog of YjeE, YdiB, also demonstrated higher ATPase activity than its dimeric form (43). The analysis of the reaction velocity showed a sigmoid dependence against the YjeE concentration. This does not necessarily indicate cooperative behavior and could for instance be due to the YjeE monomer-dimer equilibrium (Supplementary Figure S4D).

**Figure 4.**
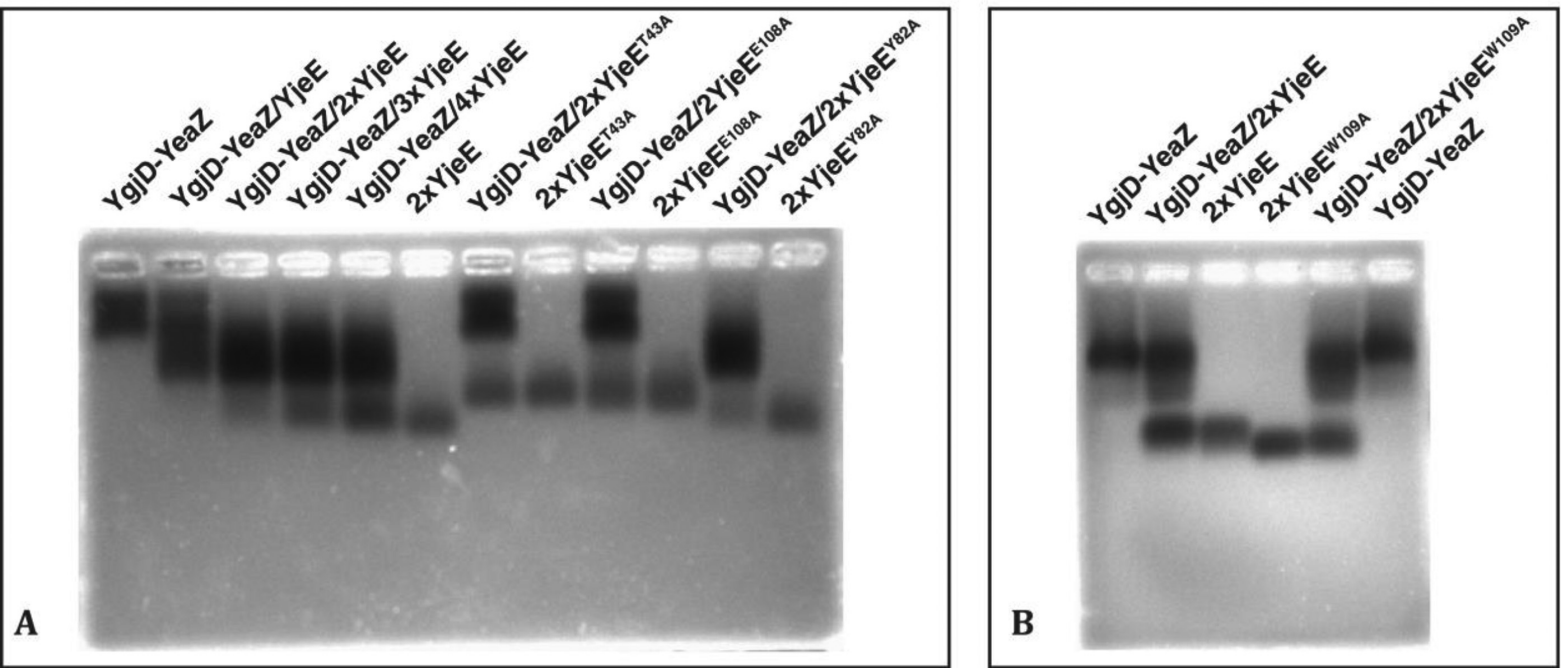
Native agarose gel shift analyses of the interaction between the YgjD–YeaZ heterodimer and YjeE. (**A**) Interaction analyses in the presence of ATP and MgCl_2_: lanes 1–6, analyses of YgjD–YeaZ at various stoichiometries of YjeE; lanes 7–12, analyses of YgjD–YeaZ in combinations with various mutants YjeE. (**B**) Interaction analyses of ADP and MgCl_2_: lanes 1–3, analysis of YgjD–YeaZ with wild-type YjeE; lanes 4–6, three analyses of YgjD–YeaZ with YjeE^W109A^ mutant.

**Figure 5.**
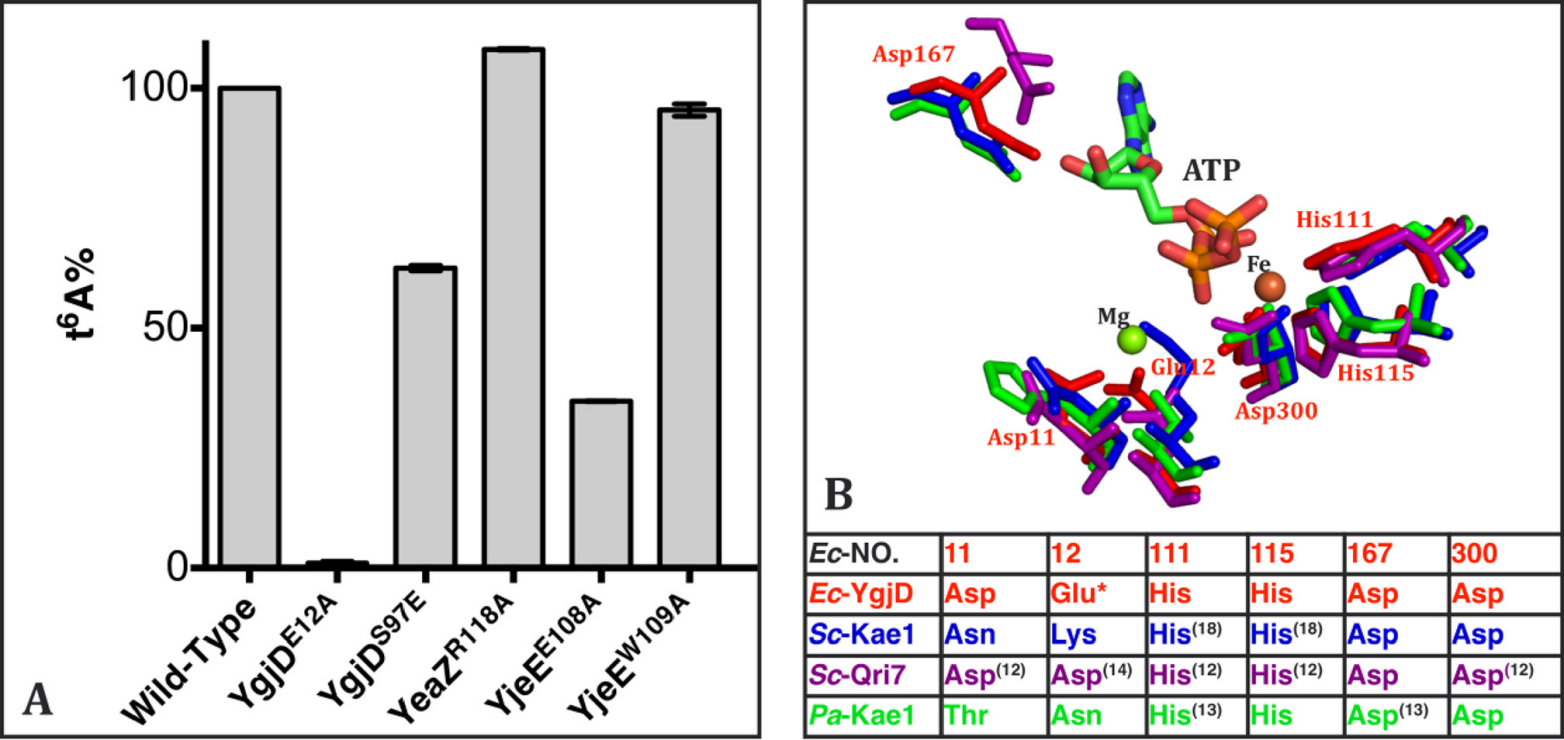
The *in vitro* biosynthesis of tRNA t^6^A by wild-type and mutant YgjD, YeaZ, YjeE and YrdC proteins. (**A**) Activities are normalized and represented as percentage of the wild-type protein activity. Only the mutated protein is included in the label. (**B**) Structural superposition of the catalytic residues of the YgjD/Kae1/Qri7 family whose mutations resulted in the abrogation of the biosynthesis of t^6^A. The structure of *Sc*–Kae1 was modeled by Phyre^2^. ATP was taken from the crystal structure of the YgjD^E12A^–YeaZ heterodimer. The Mg^2+^ ion bound at this indicated position is only observed in the crystal structure of *Ec*–YgjD. * denotes the mutation in the present study and superscript number refers to the original study on the mutations.

**Figure 6.**
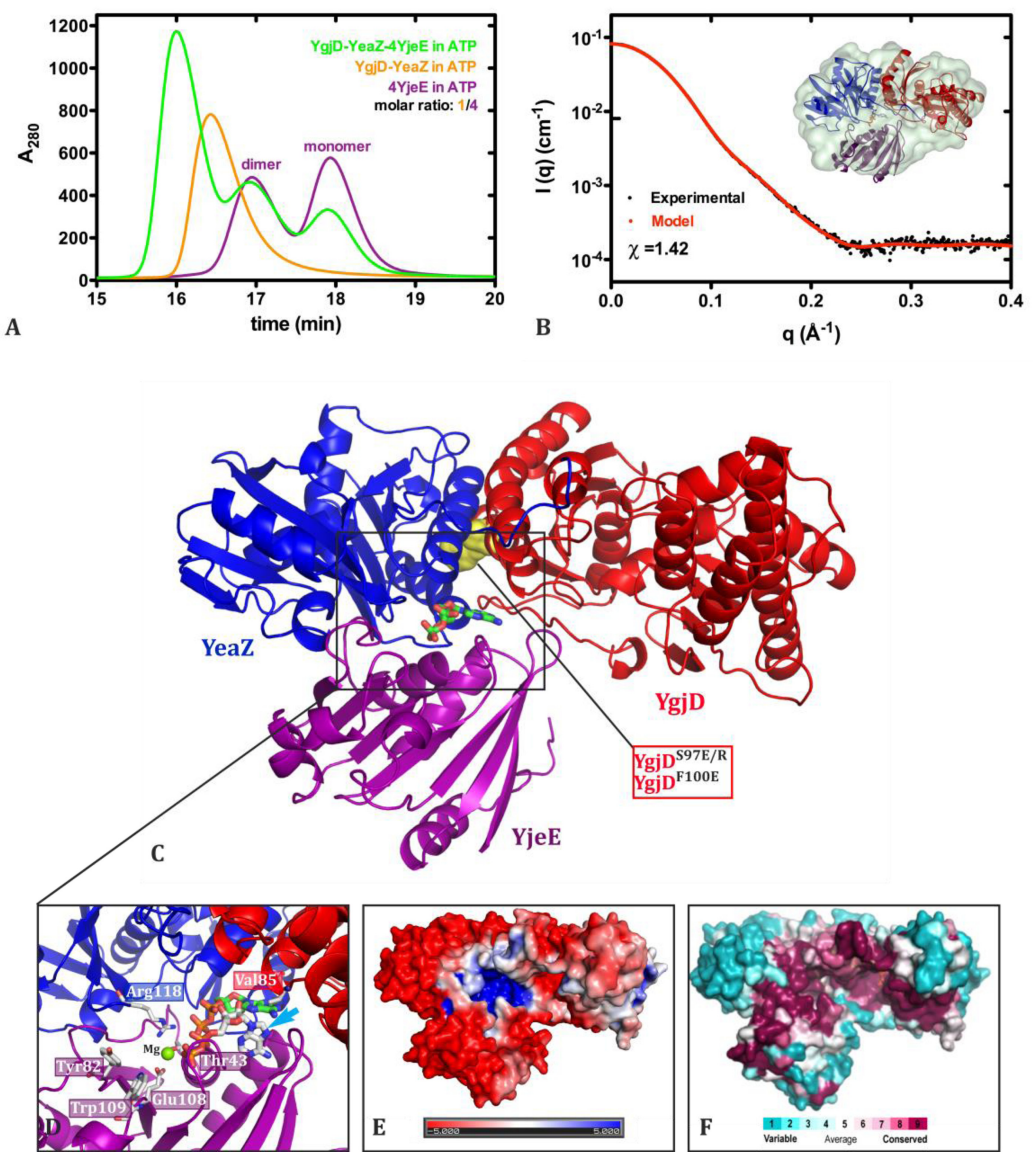
SAXS data and model of the ternary YgjD–YeaZ–YjeE complex. (**A**) The superposition of the gel filtration profiles of YgjD–YeaZ–YjeE, YgjD–YeaZ and YjeE (4× in molar ratio) in presence of 1.0 mM ATP and 2.0 mM MgCl_2_. (**B**) SAXS scattering curves of the YgjD–YeaZ–YjeE complex. The red line is the calculated scattering curve for the model represented in (**C**); the insert presents the structural model embedded in the most typical envelope extracted from the SAXS curve using the *ab initio* program GASBOR. (C) The cartoon representation of the YgjD–YeaZ–YjeE complex, the interfacial ADP is shown as sticks and interface mutation sites of YgjD are indicated in yellow spheres. (**D**) Comparison of the binding orientations of the ADP from the ternary complex YgjD–YeaZ–YjeE and the ADP (indicated by a cyan arrow) as present in the crystal structure of *Hi*–YjeE. (**E** and **F**) The sequence conservation and electrostatic potential projected on the surface of the YgjD–YeaZ–YjeE complex, adopting same orientation as in (C). The scales of the conservation and charge distribution are same as in Figure 2.

### The ATP-mediated interaction between YjeE and the heterodimer YgjD–YeaZ

Prompted by the strong activation of the ATPase activity of YjeE by YgjD–YeaZ heterodimer, we further investigated the interaction between YgjD–YeaZ and YjeE by ITC and native gel shift experiments. YgjD or YeaZ alone do not bind YjeE (Figure 1A and Supplementary Figure S1D–F), but the YgjD–YeaZ heterodimer and YjeE display a clear gel shift when combined (Figure 4A and Supplementary Figure S6C). Very importantly, in all our binding experiments we observed interaction between YgjD–YeaZ and YjeE only in presence of ATP and Mg^2+^. ITC titration of YgjD–YeaZ with YjeE revealed heat exchange signals, but the ITC curve is complex and could not be interpreted by a simple binding process, probably due to ATP hydrolysis during the titration (Supplementary Figure S5F). We therefore quantified the binding of YjeE to YgjD–YeaZ in presence of non-hydrolysable AMPPNP and Mg^2+^ (Supplementary Figure S5A). The resulting binding curve yielded a Kd of 0.60 μM, a Δ*H* of 10.16 kcal/mol and a 1:1:1 stoichiometry for the YgjD–YeaZ–YjeE complex (Table 1). The 1:1:1 stoichiometry was confirmed by varying the YjeE concentrations in the gel shift experiments (Figure 4A). We did not observe an interaction between YgjD–YeaZ and YjeE in presence of ADP (Figure 4B and Supplementary Figure S5D and E). We conclude that YjeE forms a complex with YgjD–YeaZ only in presence of ATP and that it contacts both YgjD and YeaZ.

### Structural model of the ternary complex YgjD–YeaZ–YjeE in solution

We were unable to crystallize the YgjD–YeaZ–YjeE complex in presence of ATP or AMPPNP. We then performed SAXS experiments in order to obtain structural data of the complexes in solution (Figure 6, Supplementary Figure S8, Table S2). We first analyzed the scattering curves of the individual YgjD, YeaZ, YgjD–YeaZ and YjeE components in presence of ATP and Mg^2+^. Gel-filtration and SAXS data confirmed that the isolated YgjD and YeaZ form homodimers in solution and that YgjD–YeaZ is a heterodimer. As explained in the supplementary data, the X-ray scattering curves of YgjD, YeaZ and YgjD–YeaZ can be satisfactorily described from the crystal structures using the SASREF program (Supplementary Figure S8A, S8G and S8C, respectively). The SAXS curve (Supplementary Figure S8B) of the mutant YgjD^S97E^ is compatible with a monomer showing that the mutation disrupts the dimer of YgjD (Figure 1B). The best fit was obtained by using a model with a slightly widened interface between the N- and C-terminal domains compared to the wild-type structure in complex with YeaZ. We quantified the monomer-dimer distribution of YjeE by gel filtration analysis at various time lapses. YjeE adopts an equilibrium that shifts toward the dimeric form over time and less than 20% forms monomers 18 h after purification (Figure 6A and Supplementary Figure S4C). By using a HPLC gel filtration step coupled to the SAXS measurements, we were capable of obtaining data on the separated monomeric and dimeric fractions of YjeE. The calculated scattering curve obtained from a homology model of monomeric *Ec*–YjeE (see Supplementary Materials) is in very good agreement with the scattering curve (Supplementary Figure S2D). In the case of the dimer, an envelope was obtained by using the *ab initio* program GASBOR (Supplementary Figure S2F and S2G). Two subunits of *Ec*–YjeE could be fitted into the envelope, but with only the SAXS data we could not propose a precise orientation of the subunits of the dimer. We then used gel filtration coupled to SAXS to follow the formation of the YgjD–YeaZ–YjeE complex in presence of ATP. The gel filtration profile of a mixture of YgjD–YeaZ and YjeE in a 1:4 stoichiometric ratio yielded three peaks that correspond to YgjD–YeaZ–YjeE, homodimeric and monomeric YjeE, respectively (Figure 6A). We also confirmed by SDS-polyacrylamide gelelectrophoresis the presence of YgjD, YeaZ and YjeE in a 1:1:1 molar ratio in the first peak represented in Figure 6A (data not shown). Analysis of the same protein mixture in presence of ADP showed no evidence for the formation of a stable ternary YgjD–YeaZ–YjeE complex (Supplementary Figure S8D).

The X-ray scattering data revealed that YgjD–YeaZ–YjeE has exactly the same maximal extension than YgjD–YeaZ (*D*_max_ = 90 Å, Supplementary Figure S8H) and that the envelope obtained using GASBOR (Figure 6B) is compact. These results suggest that YjeE binds at the YgjD–YeaZ interface, rather than forming a linear complex. This interpretation of the SAXS curves is compatible with biochemical data showing that only the YgjD–YeaZ heterodimer binds to YjeE and not the respective homodimers. We then attempted to create a structural model compatible with SAXS scattering curves and biochemical data. Since the presence of ATP is mandatory for the formation of the YgjD–YeaZ–YjeE complex, we reasoned that the ATP-binding site of YjeE might be involved in the interaction with YgjD–YeaZ. On the other hand, we noticed that the ADP bound at the YgjD–YeaZ interface makes contacts with both YeaZ and YgjD (Figure 2C) and that its orientation is inverted compared with the ADP bound to YjeE (Figure 6C). We therefore constructed a model of the ternary YgjD–YeaZ–YjeE complex using the SASREF program by requiring ADP of YgjD–YeaZ to be bound to YjeE in the same manner as the ADP to YjeE (PDB ID:1HTW). The calculated SAXS curve of the YgjD–YeaZ–YjeE model is in excellent agreement with the experimental scattering curve (Figure 6B). In this model, YjeE sits on top of the helical bundle formed by α2 and α3 from YgjD and α1 and α2 from YeaZ and the three partners in the complex contribute to the formation of the ATP site. YjeE therefore interacts directly both with YgjD and YeaZ. The regions surrounding the interfacial ADP site are very well conserved suggesting they are important for function (Figures 2E and 6F). The structure of the YjeE in complex with ATP is not known and there might exist significant structural rearrangements in the switch regions between the ADP and ATP bound forms.

### Exploration of structural data by site-directed mutagenesis

We carried out a number of mutations to study the structure function relationships. We summarized the strategy and results in Table 3.

**Table 3.** Summary of the mutations in this study and their functional effects

Protein	Mutation	Function	Interaction	Activity	
				ATPase	t^6^A
YgjD	E12A	Mg^2+^-coordination	Not affected	Not affected	Abolished
	S97E	Involved in the interaction between YgjD and YeaZ	Cannot form a heterodimer with YeaZ nor form a ternary complex with YeaZ and YjeE	Affected	Affected for YgjD^S97E^
	S97R				
	F100E				
	V85E	ADP^a^-coordination	Not observed in the crystal structure	Not affected	Not tested
YeaZ	Δ220–231	Interaction with YgjD	Not affected	Not affected	Not tested
	R118A	ADP^a^-coordination	Not affected	Not affected	Not tested
YjeE	T43A	ADP-coordination	ADP-binding disabled	Lost	Not tested
	E108A	Mg^2+^/ADP-coordination	ATP-binding disabled	Lost	Affected
	Y82A	Switch II	Regulation of the ATPase activity	Lost	Not tested
	W109A	Switch I	Regulation of the ATPase activity	Lost	Not affected

^a^The ADP bound in the atypical nucleotide-binding site of YeaZ at the interface of YeaZ and YgjD.

### Active site mutant of YgjD

The importance for t^6^A activity of the two conserved histidines (His^111^ and His^115^ in *Ec*–YgjD) that coordinate a Fe-ion in Kae1/Qri7 was already investigated (12,13,18). We wanted to analyze the importance of the Mg^2+^ ion, by mutating its Glu^12^ carboxylate ligand (Figure 5B) into alanine. The crystal structure of YgjD^E12A^–YeaZ in complex with ATP determined at 2.3 Å confirmed the absence Mg^2+^ in the active site. Interestingly, this mutant has ATP bound at the active site compared to ADP for the wild YgjD–YeaZ type although no binding of AMPCPP to YgjD^E12A^ was observed by ITC (Supplementary Figure S3J). YgjD probably has a very weak ATPase activity, which is inhibited in the YgjD^E12A^ mutant, explaining why we observed ATP at the active site of the mutant. Moreover, there was no electron density observed for ADP at the YgjD^E12A^–YeaZ interface, suggesting this site could have a preference for ADP rather than ATP. We then investigated the effect of this mutant on the *in vitro* biosynthesis of tRNA t^6^A. Overexpressed tRNA^Lys^ was first incubated with YgjD, YeaZ, YjeE and YrdC in presence of ATP, Mg^2+^, *L*-threonine and bicarbonate. At the end of the reaction, tRNA was enzymatically digested, the nucleosides were separated by C18 reverse phase HPLC and analyzed by mass spectrometry. As seen in Supplementary Figure S7, the presence of the modified adenine could clearly be demonstrated using wild-type proteins (Supplementary Figure S7C). We observed that the *in vitro* t^6^A activity of YgjD^E12A^ is totally abolished (Figure 5 and Supplementary Figure S7D). However the ATPase activity of the ternary complex YgjD^E12A^–YeaZ–YjeE is not affected by this mutation (Figure 3B). These data further confirm that YgjD is responsible for the t^6^A coupling reaction and YgjE for ATP hydrolysis.

### The interfacial ADP binding site

We wanted to test the pertinence of the ADP bound at the YgjD–YeaZ interface (Figure 2C) using two mutants, YeaZ^R118A^ and YgjD^V85E^. The crystal structure of YgjD^V85E^–YeaZ in the presence of ATP showed no ADP-like electron density at the dimer interface. Nonetheless, YgjD–YeaZ^R118A^ still interacts with YjeE in presence of AMPPNP, as determined by ITC (Table 1 and Supplementary Figure S5C). The ATPase activity of the ternary YgjD^V85E^–YeaZ–YjeE and YgjD–YeaZ^R118A^–YjeE complexes remained unaffected (Figure 3B) and the latter is fully active for the *in vitro* biosynthesis of t^6^A (Figure 5A).

### The YgjD–YeaZ interface

In order to estimate the importance of heterodimer formation, we generated mutations at the YgjD–YeaZ interface. Ser^97^ and Phe^100^ of YgjD are both involved in packing of the helices and are surrounded by hydrophobic residues (Figure 6C). ITC, gel filtration and native gel data showed that YgjD^S97E^, YgjD^S97R^ and YgjD^F100E^, are no longer capable of forming homodimers nor heterodimers with YeaZ (Table 1 and Supplementary Figure S1C for YgjD^S97E^ by ITC and Supplementary Figure S6A and S6C for YgjD^S97E^, YgjD^S97R^ and YgjD^F100E^ by gel filtration and native gel shift, respectively), suggesting that the same regions of YgjD are involved both for homo- and heterodimer formation. We demonstrated by ITC and native gel shift that a mixture of YgjD^S97R^ and YeaZ is not able to associate with YjeE in presence of AMPPNP or ATP (Supplementary Figures S5I and S6C). None of these variants are capable of activating the ATPase activity upon mixing with YeaZ and YjeE (Figure 3B), suggesting that the formation of the YgjD–YeaZ heterodimer is mandatory for the ATPase activation of YjeE. However, the *in* *vitro* t^6^A activity of YgjD^S97E^ decreased by only ∼40% (Figure 5A and Supplementary Figure S7E).

### YjeE ATP binding site mutations

YjeE displays structural features typical for GTPase proteins (34,35), characterized by the presence of switch regions that undergo conformational changes upon nucleotide hydrolysis, triggering signal transduction to other proteins or nucleic acids (42). Two such switch regions were identified in the structure of YjeE: switch I is involved in coordination of the Mg^2+^ ion and switch II is in proximity to the β- and γ- phosphates of bound nucleotide (Supplementary Figure S2E). To further explore the role of YjeE we designed two types of mutations: those with the goal of affecting nucleotide binding and ATPase activity (residues Thr^43^ and Glu^108^) and mutations of residues in the putative switch regions (Trp^109^ and Try^82^). Tyr^82^ from switch II is in a connection between β3 and β4 strands and should be close to the γ-phosphate of ATP whereas Trp^109^ from switch I is next to the Mg^2+^-binding residue Glu^108^ and stacks against Tyr^82^ (Supplementary Figure S2E). The ATPγS- or ADP-binding properties of these mutants were quantified by ITC. While YjeE^Y82A^ and YjeE^W109A^ still bound to ATPγS and ADP with a Kd of 8.90 and 4.83 μM, respectively (Table 2 and Supplementary Figure S3E and F), the binding of ADP to both YjeE^T43A^ and YjeE^E108A^ was abrogated (Supplementary Figure S3K and S3L). The ATPase activity of the YjeE^Y82A^ has dropped to 40% and that of the other mutants fell below 10% compared to the wild-type YjeE (Figure 3B). These data strengthen the hypothesis that YjeE is the *bona fide* ATPase activated by the YgjD–YeaZ heterodimer.

We further tested whether these mutants are capable of forming a complex with YgjD–YeaZ. Native gel shift experiments (Figure 4A) showed that the YjeE^T43A^ and YjeE^E108A^ mutants no longer interact with YgjD–YeaZ while the binding capacity of the switch mutants YjeE^Y82A^ and YjeE^W109A^ remained unaffected. The capacity of YjeE to bind ATP therefore seems to be correlated with its binding to YgjD–YeaZ. ITC measurements further showed that the binding capacity of YjeE^W109A^ to YgjD–YeaZ is comparable to that of wild-type YjeE in presence AMPPNP (Table 1 and Supplementary Figure S5B). The X-ray scattering curve of the YgjD–YeaZ–YjeE^W109A^ mixture is identical to that of the wild-type complex, suggesting that its overall structure is not affected (Supplementary Figure S8F).

We then wanted to find out if the ATPase activity of YjeE is essential for the *in vitro* biosynthesis of tRNA t^6^A. Interestingly, although the YjeE^E108A^ and YjeE^W109A^ mutants no longer exhibit ATPase activity (Figure 3B), the *in vitro* biosynthesis of t^6^A by YjeE^W109A^ remained unaffected while it dropped by 60% for YjeE^E108A^ (Figure 5A and Supplementary Figure S7). We conclude that for the *in vitro* biosynthesis of tRNA t^6^A the ATPase activity of the ternary complex YgjD–YeaZ–YjeE seems dispensible.

### Deletion of the C-terminal region of YeaZ

As the C-terminus of YeaZ (220–231) is not observed in the crystal structure of the YeaZ homodimer but is well-positioned by interaction with YgjD in the crystal structure of the YgjD–YeaZ heterodimer (Figure 2A), truncated YeaZ^1–219^ was produced to test the contribution of the C-terminal YeaZ^220–231^ in formation of heterodimer YgjD–YeaZ. Binding was followed by ITC and native gel shift and it was demonstrated that YeaZ^1–219^ still interacts with YgjD with slightly higher affinity (Kd of 34 nM compared to 122 nM for intact YeaZ) (Supplementary Figure S6B, Table 1 and Supplementary Figure S1B). The X-ray scattering curve for the YgjD–YeaZ^1–219^–YjeE complex was identical to that of YgjD–YeaZ–YjeE (Supplementary Figure S8E), confirming the C-terminal tail of YeaZ^220–231^ is not essential for the formation of the ternary complex.

## DISCUSSION

### The YgjD–YeaZ heterodimer is essential for activity

The reaction scheme of the biosynthesis of tRNA t^6^A in bacteria is composed of two main steps. In the first half of the reaction, YrdC utilizes ATP, *L*-threonine and bicarbonate to synthesize an unstable intermediate TCA, whose threonylcarbamoyl-moiety is subsequently transferred onto the adenosine at position 37 of the substrate tRNAs by the cooperative action of three proteins YeaZ, YgjD and YjeE. The yeast mitochondrial ortholog of YgjD, Qri7, is capable of transferring the threonylcarbamoyl-moiety of TCA onto tRNA without assistance of other protein partners (12). This provides strong evidence that YgjD is responsible for the coupling reaction while YeaZ and YjeE assist its action (11,12,14). How exactly YeaZ and YjeE contribute to the tRNA t^6^A modification *in vivo* remains unknown. We showed here that *E. coli* YgjD forms a stable heterodimer with YeaZ, and that this dimer forms a complex with YjeE in presence of ATP, confirming previous studies on the *Salmonella thyphimurium* orthologs (24). The YgjD–YeaZ interface is very similar to that of the YeaZ and Qri7 homodimers (12,41). Mutations disrupting the YgjD–YeaZ heterodimer abrogated interaction with YjeE and are largely inactive in the *in vitro* biosynthesis of tRNA t^6^A. A similar dependence of the t^6^A biosynthesis upon homodimerization was observed for Qri7 (12). Interestingly we observed that YgjD alone also forms homodimers but these are unable to bind YjeE and are inactive. The YgjD^S97E^, YgjD^S97R^ and YgjD^F100E^ mutants neither form homodimers nor heterodimers with YeaZ, suggesting the YgjD homodimer is structurally similar to those of Qri7 and YeaZ. Despite the similarities between the interaction modes of YgjD, YeaZ and Qri7, the complementation of *E. coli ygjD* requires both the *ygjD* and *yeaZ* orthologs from *B. subtillus*, showing a species-specific recognition between YgjD by YeaZ (10).

The coupling of TCA to A_37_ of cognate tRNA in archaea and yeast is carried out by the KEOPS complex that consists of a linear arrangement of the Kae1, Bud32, Cgi121 and Pcc1 proteins (16,21). Pcc1 is required for the *in vitro* biosynthesis of tRNA t^6^A in both archaea and yeast cytoplasm (12,13,18). In the structural model of archaean KEOPS, Pcc1 binds to the N-terminal lobe of Kae1 whereas Bud32 binds to the C-terminal lobe. Pcc1, although unrelated to YeaZ, forms a complex with Kae1 whose interface is structurally mimicking that of YgjD–YeaZ (Figure 2H). The binding of a protein partner (Qri7, Pcc1 or YeaZ) to the N-terminal lobe of Qri7/Kae1/YgjD seems to be essential for t^6^A activity and is likely involved in the binding of the tRNA substrate. We were not able to detect the binding of tRNA to the YgjD–YeaZ–YjeE complex nor to any of the individual proteins by electrophoretic mobility shift assay (data not shown), meaning that the tRNA binding is too weak to be detected and occurs only transiently.

### The ATPase YjeE is strongly activated by YgjD–YeaZ

The role of the essential YjeE protein in the transfer of threonylcarbamoyl-moiety from TCA onto A_37_ of tRNA in bacteria remains unknown (11). Previous two hybrid data showed that YgjD, YeaZ and YjeE form a network and that the interaction between YeaZ and YjeE was mutually exclusive with the formation of the YgjD–YeaZ complex. *S. typhimurium* YjeE was reported to bind both to YgjD–YeaZ and to YgjD in presence of ADP or ATP (25). Our experiments however demonstrate that only the YgjD–YeaZ heterodimer strongly interacts with YjeE and that this interaction is dependent on the presence of ATP but not ADP. The resemblance *Hi*–YjeE with small GTPases suggested that YjeE might work as a molecular switch triggered by ATP hydrolysis (36). We showed that *Ec*–YjeE binds more strongly to ADP than to ATPγS or AMPPNP and that its intrinsic weak ATPase activity is strongly activated by binding to YgjD–YeaZ (14,34). Mutants of YgjD (YgjD^S97E^, YgjD^S97R^ or YgjD^F100E^) that are unable to form heterodimers with YeaZ are incapable of activating the ATPase activity of YjeE. The YjeE^W109A^ (in switch I) and YjeE^Y82A^ (in switch II) mutations did not affect ATP or YgjD–YeaZ binding, but they lost their ATPase activity by 100 and 65% respectively. Interestingly, the *in vitro* t^6^A biosynthesis of the YjeE^W109A^ mutant remained intact. The YjeE^T43A^ and YjeE^E108A^ mutations abrogated ADP- or ATP-binding and hence they were not able to interact with YgjD–YeaZ. The activity of YjeE^E108A^ in biosynthesis of tRNA t^6^A is reduced by more than 60%. The binding capacity of YjeE to YgjD–YeaZ rather than its ATPase activity seems to be primordial for tRNA t^6^A modification. This confirms the observation that the *B. subtilis* YgjD, YeaZ and YjeE orthologs function in biosynthesis of tRNA t^6^A in absence of ATP (11). However the ATPase activity of YjeE is necessary for cell growth since it was observed that the ATPase inactive K41A mutant of the *B. subtilis* ortholog YdiB had the same negative effect on cell growth than the knockout mutant (43). This suggests that YjeE may be involved in other important cellular processes.

### ATP-mediated formation of YgjD–YeaZ–YjeE

Our biochemical and structural data suggest that YjeE contacts both YgjD and YeaZ to form a compact heterotrimeric complex. The YgjD–YeaZ interface contains a well-conserved surface patch that is centered on a bound ADP molecule. We used this ADP moiety as an anchor for docking the YjeE protein onto the YgjD–YeaZ interface. The resulting model of the ternary complex brings into light many interesting features. First, the interfacial ADP-binding site is composed of residues from the three proteins and may explain theATPase activating effect of YjeE upon interaction with YgjD–YeaZ. Second, YjeE interacts both with YeaZ and YgjD, as expected for its binding specificity for heterodimeric YgjD–YeaZ. Third, the active site of YjeE binds to well-conserved surface patches of the YgjD–YeaZ complex. Fourth, in our model of the ternary complex compatible with the SAXS data, YjeE and YgjD form a crater large enough to accommodate the substrate tRNA. The catalytic metal site of YgjD is at the bottom of this crater. YjeE is therefore well positioned in the ternary complex to be involved in substrate tRNA binding. The rigid superposition that was used for the construction of the model of the ternary complex created some small steric clashes between Ala^130^ and Gln^131^ from YjeE and Pro^327^ and Arg^328^ of YgjD, and between Pro^61^–Thr^64^ of YjeE and Gly^120^ and Ala^169^ of YeaZ, all of which reside in potentially flexible loops. It should be noticed that the YgjD–YeaZ–YjeE complex forms in the presence of ATP but that the complex was modeled using the structure of the YjeE–ADP complex. Small G proteins usually adopt rather different conformations between the GDP and GTP bound forms. It seems therefore plausible that the ATP bound form of YjeE may undergo conformational changes in the switch regions that might relieve the present steric clashes in our model. The mutations of two residues that contact this ADP molecule (YeaZ^R118A^ and YgjD^V85E^) did not confer any effect on the heterodimerization of YgjD and YeaZ, nor on the activation of the ATPase activity of YjeE. The intact t^6^A biosynthesis but corrupted ATPase activities of the YjeE^W109A^ mutant showed that formation of the ternary YgjD–YeaZ–YjeE complex is primordial.

## CONCLUSION

All organisms use Sua5/YrdC for the biosynthesis of the intermediate TCA, but the bacteria, eukaryotes/archaea and eukaryotic mitochondria developed different strategies for the condensation of threonylcarbamoyl-moiety from TCA onto adenosine at position 37 of tRNA. Although these strategies involve different proteins, they have several points in common. All the tRNA t^6^A modification systems use a closely related threonylcarbamoyl-transferase, Qri7/Kae1/YgjD, sharing a common structure and active site configuration, centered on a very conserved metal cluster. These enzymes all need support from a protein subunit bound to the N-terminal lobe. Although, the structures of these subunits are different for Qri7/Kae1/YgjD, the interfaces between the subunits are almost identical. The most plausible hypothesis about the function of non-catalytic subunits is their involvement in tRNA recognition, but few data are available on tRNA interaction for any of the t^6^A biosynthesis systems. Kae1 and YgjD further need Bud32/Cgi121/Pcc1/(Gon7) and YeaZ/YjeE to carry out their activity in biosynthesis of t^6^A, respectively. Although Bud32 is annotated as a protein kinase, structural and functional studies and comparison with its paralog RIO kinase led to the hypothesis that Bud32 may act as an ATPase within the KEOPS complex rather than as a protein kinase. RIO kinase plays an essential role in pre-40S ribosomal subunit maturation and its ATP hydrolysis triggers 40S subunit biogenesis. YjeE and Bud32 probably play regulatory roles in the biosynthesis of tRNA t^6^A through their interactions with and/or hydrolysis of ATP. YjeE and Bud32 are unrelated proteins and they interact very differently with YgjD and Kae1, respectively. Further studies will have to establish the functional relationships of these proteins within the t^6^A biosynthesis system. The crystal structure of the ternary complex YgjD–YeaZ–YjeE in complex with tRNA will also shed light on more complete mechanistic understanding of the transfer of threonylcarbamoyl-moiety from TCA onto A_37_ of tRNA.

## ACCESSION NUMBERS

PDB IDs: 4WOS, 4WQ4 and 4WQ5.

## SUPPLEMENTARY DATA

Supplementary Data are available at NAR Online.

SUPPLEMENTARY DATA
