# Supplementary material for: The ATP-mediated formation of the YgjD–YeaZ–YjeE complex is required for the biosynthesis of tRNA t6A in Escherichia coli
Source: Nucleic Acids Res. 2015 Jan 10;43(3):1804–17. doi: 10.1093/nar/gku1397 (PMC4330362; doi:10.1093/nar/gku1397)
Supplement: SUPPLEMENTARY DATA [file supp_43_3_1804__index.html]

The ATP-mediated formation of the YgjD–YeaZ–YjeE complex is required for the biosynthesis of tRNA t6A in Escherichia coli — The ATP-mediated formation of the YgjD–YeaZ–YjeE complex is required for the biosynthesis of tRNA t6A in Escherichia coli — SUPPLEMENTARY DATA 

# The ATP-mediated formation of the YgjD–YeaZ–YjeE complex is required for the biosynthesis of tRNA t6A in *Escherichia coli*

## SUPPLEMENTARY DATA

**Files in this Data Supplement:**

- SUPPLEMENTARY DATA
