# Supplementary material for: The ATP-mediated formation of the YgjD–YeaZ–YjeE complex is required for the biosynthesis of tRNA t6A in Escherichia coli
Source: Nucleic Acids Res. 2015 Jan 10;43(3):1804–17. doi: 10.1093/nar/gku1397 (PMC4330362; doi:10.1093/nar/gku1397)
Supplement: SUPPLEMENTARY DATA [file supp_gku1397_nar-03149-h-2014-File003.pdf]

SUPPLEMENTARY Material

Supplementary experimental

Primers in mutagenesis

The mutations and primers are listed in the table below.

Mutation	Primer	Sequence (5'>3')
**YgjD-F100E**	YgjD-100E-fw	ggggcgttctctggcggaagcctgggacgttccggc
	YgjD-100E-rv	gccggaacgtcccaggcttccgccagagaacgcccc
**YgjD-S97E**	YgjD-S97E-fw	gcgcgaccgtggggcgtgaactggcgtttgcctggga
	YgjD-S97E-rv	tcccaggcaaacgccagttcacgccccacggtcgcgc
**YgjD-S97R**	YgjD-S97R-fw	cgcgaccgtggggcgtcgtctggcgtttgcctggg
	YgjD-S97R-rv	cccaggcaaacgccagacgacgccccacggtcgcg
**YgjD-E12A**	YgjD-E12A-fw	gaaacttcctgcgatgcaaccggcatcgccatt
	YgjD-E12A-rv	aatggcgatgccggttgcatcgcaggaagtttc
**YgjD-V85E**	YgjD-V85E-fw	ccgcaggccctggattagaaggcgcgctactg
	YgjD-V85E-rv	cagtagcgcgccttctaatccagggcctgcgg
**YeaZ-Δ220**	YeaZ-del220-fw	gaaccggtttatttacgtaactaagtcgcatggaagaaacttccg
	YeaZ-del220-rv	cggaagtttcttccatgcgacttagttacgtaaataaaccggttc
**YeaZ-R118A**	YeaZ-R118A-fw	ggcagccattgacgcggcaatgggcgaagtttac
	YeaZ-R118A-rv	gtaaacttcgcccattgccgcgtcaatggctgcc
**YjeE-E108A**	YjeE-E108A-fw	catctgcctggtggcgtggccacaacaag
	YjeE-E108A-rv	cttgttgtggccacgccaccaggcagatg
**YjeE-T43A**	YjeE-T43A-fw	tggcgatttaggcgcaggtaaagccacctttagcc
	YjeE-T43A-rv	ggctaaaggtggctttacctgcgcctaaatcgcca
**YjeE-Y82A**	YjeE-Y82A-fw	ttaatggtctatcactttgatttggcccgccttgccgatcc
	YjeE-Y82A-rv	ggatcggcaaggcgggccaaatcaaagtgatagaccattaa
**YjeE-W109A**	YjeE-W109A-fw	catctgcctggtggaggcgccacaacaaggtaca

	YjeE-W109A-rv	tgtaccttgttgtggcgcctccaccaggcagatg

Expression and purification of the recombinant proteins

Recombinant YgjD (TsaD), YeaZ (TsaB), YjeE (TsaE), YrdC (TsaC) and their mutants were heterologously expressed in *E. coli* BL21(DE3) cells (Invitrogen). Conventionally, cells were grown in 2YT medium (Life Technology, Inc.) supplemented with proper antibiotics at 37 °C to OD_{600} ~0.6. Protein expression was induced by addition of 1.0 mM isopropyl β-D-1-thiogalactopyranoside for 3 hours at 37 °C. Cells were then harvested by centrifugation and re-suspended in lysis buffer (20 mM Tris-HCl pH 7.5, 200 mM NaCl), stored at -20 °C until purification.

Cells were thawed in a water bath at ambient temperature and lysed by sonication in ice water. The cell lysates were centrifuged for 30 min at 12000 g to remove the cell debris and the supernatant was applied to a Ni-NTA resin for purification, followed by successive ion exchange and size exclusion chromatographic steps. The purity of proteins after each purification step was analyzed by SDS-PAGE. After size exclusion chromatography protein fractions with purity greater than 95% in preparative buffer (20 mM HEPES pH 7.5, 200 mM NaCl and 5 mM 2-mercaptoethanol) were collected and stored at -80 °C by flash-freezing in liquid nitrogen. YgjD, YeaZ, YrdC, YjeE and their variants were purified by affinity chromatography using NiNTA resins, followed by Q-anion exchange chromatography and size exclusion chromatography.

Isothermal Titration Calorimetric (ITC) assay

ITC measurements for the determination of the thermodynamic parameters of protein binding equilibrium were performed with MicroCalITC200 (GE Healthcare). For each measurement a total of 20 injections of 2 μL injectant were performed at intervals of 180 seconds under continuous stirring. Normalized area data in kcal/mol of each integrated injected peak were plotted versus the molar ratio of injectant to sample titrated using the Origin 7.0 Plotting Software (MicroCal Inc.)

Crystallization

The individually purified YgjD and YeaZ were mixed at a molar ration of 1:1 and the mixture was subjected to size exclusion chromatography in buffer containing 20 mM HEPES pH 7.5, 200 mM NaCl and 5 mM 2-mercaptoethanol. Fractions

containing the YgjD-YeaZ heterodimer as visualized by SDS-PAGE gel were pooled and concentrated to ~10.0 mg/ml. We only obtained good crystals of the YgjD-YeaZ heterodimer from crystallization conditions that contain ATP or ADP. Thin plate crystals of the heterodimer YgjD-YeaZ were obtained at 293K by hanging-drop vapor diffusion from a crystallization solution consisting of 4.5 mg/ml YgjD/YeaZ heterodimer, 60 mM HEPES pH 7.5, 100 mM NaCl, 15% PEG4000, 150 mM Lithium Acetate, 2.5 mM ATP-Mg^{2+} and 2.5 mM *L*-threonine. The same purification and crystallization procedures were applied to the mutants. The crystals of the heterodimers $YgjD^{E12A}$-YeaZ and $YgjD^{V85E}$-YeaZ were obtained under similar conditions. The crystals were cryoprotected in the reservoir solution supplemented with ~25% Ethylene Glycol and flash-cooled and stored in liquid nitrogen until data collection.

Data collection and structure determination

Data collection was carried out on beamlines Proxima1 and Proxima2 at the SOLEIL Synchrotron (Saint-Aubin, France). Data were processed, integrated and scaled with the XDS program package (1). The isomorphous crystals of the YgjD-YeaZ heterodimer and their mutants, $YgjD^{E12A}$-YeaZ and $YgjD^{V85E}$-YeaZ, belonged to space group P1 with unit cell parameters of a= 63.62 Å, b= 68.48 Å, c= 87.07 Å, α=109.38°, β=92.66° and γ=117.66°. The structure of the heterodimer YgjD-YeaZ was solved by molecular replacement using the PHASER module from the CCP4 package starting with search models of *P. abyssi* Kae1 (PDB ID: 2IVP) and *E. coli* YeaZ (PDB ID: 1OKJ) (2-4). The Phenix AutoBuild was employed to build the flexible regions in the initial model after molecular replacement (5), followed by interactive and manual building using the COOT (6). The final model was refined to satisfactory values using Refmac5 (Table S1) (7). Two copies of the YgjD-YeaZ heterodimer were found in an asymmetric unit. ADP or ATP molecules and Mg^{2+} ions were modeled into the residual *Fo-Fc* electron density contoured at 3.0 σ. Additionally, fluorescence measurements on the crystals at Fe-edge wavelength confirmed $Fe^{2+/3+}$ ion was present in the YgjD structure (data not shown). Finally, water molecules were added manually to the protein model and validated by refinement. The Ramachdran plot analysis was performed with PROCHECK (8). Data collection and structure refinement statistics are summarized in Table S1.

Small Angle X-ray Scattering (SAXS) experiment

SAXS experiments were carried out on the SWING beamline at the SOLEIL synchrotron radiation facility (Saint-Aubin, France). The sample to detector (Aviex CCD) distance was set to 1500 mm, allowing reliable data collection over the momentum transfer range 0.008 $Å^{-1} < q < 0.5 Å^{-1}$ with $q=4\pi\sin\theta/\lambda$ where 2θ is the scattering angle and λ the wavelength of the *X*-rays (λ= 1.0 Å). In order to separate the isolated partners from complexes, SAXS data were collected on solutions eluting from the on line size-exclusion high-performance liquid chromatography (SE-HPLCBio-SEC3 Agilent) column available on SWING and directly connected to the SAXS measuring cell. The purified YgjD, YeaZ, the YgjD-YeaZ heterodimer, YjeE and their mutants were premixed in absence of any added nucleotides or with 1 mM ATP or ADP and 4 mM $MgCl_2$. The individual proteins or mixtures of them were injected in the column pre-equilibrated with preparative buffer (20 mM HEPES pH 7.5, 200 mM NaCl and 5 mM 2-mercaptoethanol) without addition of any nucleotides or supplemented with 1 mM ATP or ADP and 4 mM $MgCl_2$. Flow rate was 200 µL/min, frame duration was 1 s and the dead time between frames was 0.5 s. For each frame, the protein concentration (between 0.5 and 2 mg/mL at the top of elution peak) was estimated from UV absorption at 280 nm and 295 nm using a spectrometer located immediately upstream of the SAXS measuring cell. Due to the presence of ATP or ADP, the concentration of the protein could not be determined accurately.. Selected identical frames corresponding to each elution peak were averaged. A large number of frames were collected before the void volume and averaged to account for buffer scattering. SAXS data were normalized to the intensity of the incident beam and background (i.e. the elution buffer) subtracted using the programs FoxTrot (courtesy of SWING beamline) and Primus (9,10). The scattered intensities were displayed on an absolute scale using the scattering by water. The molar mass M of the scattering objects is usually calculated from zero-angle scattered intensity *I(0)* which is proportional to M and to the concentration. This method cannot be applied in our case because of the uncertainties in concentration. In order to determine unambiguously the oligomeric state of the protein or complex the molar mass was obtained using the macromolecule volume and the SAXS-MoW method available at http://www.if.sc.usp.br/~saxs/. The model of YjeE was built by Phyre[2] using the crystal structure of *H. influenzae* YjeE (PDB ID: 1HTW) in which the relative coordinates of the ADP, Mg^{2+} and water molecules were modelled to YjeE (11,12).

The general strategy to analyze the SAXS data consists of using the crystal structures of the partners and determining the quaternary structure using the program SASREF. The program runs typically twenty times and the model that gives the best adjustment to the experimental curve is chosen. The few residues missing in the C-terminal parts of the proteins are modelled using the program MODLOOP. An ultimate adjustment is performed using the program CRYSOL. Only when the information on the relative position of the different partners is absent –which was the case for the YjeE dimer and for the YgjD-YeaZ-YjeE complex - a preliminary step is added: the envelope of the macromolecule is deduced from the I(q) curve using the program GASBOR in order to guide the building of the complex.

The structure of the $YgjD^{S97E}$ monomer was extracted from the crystal structure of the heterodimer YgjD-YeaZ determined in this study. The two domains of the monomer were subsequently slightly moved using SASREF in order to fit to the experimental curve. The homodimer YgjD was constructed by superimposition on the crystal structure of YgjD-YeaZ followed by a very slight opening of each monomer. The SAXS curve of the heterodimer YgjD-YeaZ was perfectly described using the crystal structure. The same holds true for the homodimer of YeaZ that was well described using the crystal structure (PDB ID: 1OKJ) with the missing C-ter parts built by PHYRE2 and moved using SASREF. The model of *Ec*-YjeE was built by $Phyre^2$ using the crystal structure of *H. influenzae* YjeE (PDB ID: 1HTW) in which the relative coordinates of the ADP and Mg^{2+} molecules were conserved. This model gives a perfectly adjustment to the experimental curve. The modelization of the ternary complex is described in the main text.

In vitro assay of biosynthesis of tRNA t^6A and digestion of tRNA

The 25 μg *E.coli* $tRNA^{Lys}$ was incubated with 2.5 μM wild-type proteins (YgjD, YeaZ, YjeE and YrdC) or their mutants in 80 μl of assay buffer that contained 50 mM Tris pH 7.4, 200 mM NaCl, 10 mM $MgCl_2$, 25 mM KCl, 4mM *L*-threonine, 2 mM ATP and 10 mM sodium bicarbonate. The reactions were proceeded for 90 min at 37 °*C* and terminated by heating the reaction mixture to 95 °*C* for 5 min, followed by fast cooling-down in ice water. tRNA was unfolded and the protein precipitates were removed by centrifugation. The tRNA was further enzymatically digested with P1 nuclease, phosphodiesterase and alcaline phosphatase (All products from Sigma), following the instructions of the products and a method developed for analysis of

nucleosides by HPLC (13). The digested nucleosides were applied to HPLC and LC/MS for analysis. The production of *L*-threonylcarbamoyladenylate (TCA) was done by incubation of 5 μM YrdC for 30 min at 37°C in the assay buffer containing 50 mM Tris pH 7.4, 200 mM NaCl, 10 mM $MgCl_2$, 25 mM KCl, 8 mM L-threonine, 4 mM ATP and 20 mM sodium bicarbonate. The YrdC was removed by applying this reaction mixture to a centrifugal concentrator with a molecular weight cut-off of 5 KD (VWR). The eluate containing the TCA was obtained by spinning the concentrator of for 5 min at 13000g at 8 *°C*. The stability of TCA was monitored by HPLC over time (data not shown).

HPLC and MS analysis of the t^6A

100 μl reaction mixture was applied to HPLC using a μRPC C2/C18 ST 4.6/100 column with a pore size of 120 Å (Amersham Biosciences Inc.) and nucleosides were detected by UV at 258 nm. The column was equilibrated with buffer A (10 mM Ammonium Acetate, pH 7.0). The gradient used for elution was 100% buffer A from 0 to 8 min followed by a linear gradient of 0-100% buffer B (6 mM Ammonium Acetate, pH 7.0 and 40% acetonitrile) from 8 to 30 min. The standard Cytosine (C), Uracil (U), Guanine (G), Adenine (A) (Sigma) and t^6A (kindly provided by R.W. Adamiak, Polish Academy of Sciences) were used as reference for determination of the elution time of each digested nucleosides in the reaction mixture (14). The area under each peak that corresponds to C, U, G, A and t^6A was used for quantification of the digested nucleosides of tRNA. The percentage of t^6A is calculated as the average of area of t^6A to area of C, U and G, respectively, normalized to tRNA alone and tRNA modified by wild type YgjD, YeaZ, YjeE and YrdC. MS analyses of nucleosides were performed by mass spectrometer Micro TofQ (BRUKER DALTONICS) equipped with HPLC (Ultimate3000, DIONEX) using the μRPC C2/C18 ST 4.6/100 column. The gradient used in LC/MS was 100% buffer A from 0 to 8 min followed by a linear gradient of 0-100% buffer B (6 mM Ammonium Acetate, pH 7.0 and 40% acetonitrile) from 8 to 30 min. MS was employed to confirm the masses of C, U, G, A and t^6A from digested nucleosides.

References

1. Kabsch, W. (2010) Xds. *Acta Crystallogr D Biol Crystallogr*, **66**, 125-132.
2. Winn, M.D., Ballard, C.C., Cowtan, K.D., Dodson, E.J., Emsley, P., Evans, P.R., Keegan, R.M., Krissinel, E.B., Leslie, A.G., McCoy, A. *et al.* (2011) Overview of the CCP4 suite and current developments. *Acta Crystallogr D Biol Crystallogr*, **67**, 235-242.
3. Hecker, A., Leulliot, N., Gadelle, D., Graille, M., Justome, A., Dorlet, P., Brochier, C., Quevillon-Cheruel, S., Le Cam, E., van Tilbeurgh, H. *et al.* (2007) An archaeal orthologue of the universal protein Kae1 is an iron metalloprotein which exhibits atypical DNA-binding properties and apurinic-endonuclease activity in vitro. *Nucleic Acids Res*, **35**, 6042-6051.
4. Stelter, M., Molina, R., Jeudy, S., Kahn, R., Abergel, C. and Hermoso, J.A. (2014) A complement to the modern crystallographer's toolbox: caged gadolinium complexes with versatile binding modes. *Acta Crystallogr D Biol Crystallogr*, **70**, 1506-1516.
5. Adams, P.D., Afonine, P.V., Bunkoczi, G., Chen, V.B., Davis, I.W., Echols, N., Headd, J.J., Hung, L.W., Kapral, G.J., Grosse-Kunstleve, R.W. *et al.* (2010) PHENIX: a comprehensive Python-based system for macromolecular structure solution. *Acta Crystallogr D Biol Crystallogr*, **66**, 213-221.
6. Emsley, P. and Cowtan, K. (2004) Coot: model-building tools for molecular graphics. *Acta Crystallogr D Biol Crystallogr*, **60**, 2126-2132.
7. Winn, M.D., Murshudov, G.N. and Papiz, M.Z. (2003) Macromolecular TLS refinement in REFMAC at moderate resolutions. *Methods Enzymol*, **374**, 300-321.
8. Laskowski, R.A., MacArthur, M.W., Moss, D.S. and Thornton, J.M. (1993) PROCHECK - a program to check the stereochemical quality of protein structures. *Journal of Applied Crystallography*, **26**, 283-291.
9. Konarev, P.V., Petoukhov,M.V., Volkov,V.V. and Svergun,D.I. . (2006) ATSAS 2.1, a program package for small-angle scattering data analysis. *J. Appl. Cryst.*, **39**, 277-286.
10. Konarev, P.V., Volkov, V.V., Sokolova, A.V., Koch, M.H.J. and Svergun, D.I. (2003) PRIMUS: a Windows PC-based system for small-angle scattering data analysis. *Journal of Applied Crystallography*, **36**, 1277-1282.
11. Kelley, L.A. and Sternberg, M.J. (2009) Protein structure prediction on the Web: a case study using the Phyre server. *Nature protocols*, **4**, 363-371.
12. Teplyakov, A., Obmolova, G., Tordova, M., Thanki, N., Bonander, N., Eisenstein, E., Howard, A.J. and Gilliland, G.L. (2002) Crystal structure of the YjeE protein from Haemophilus influenzae: a putative Atpase involved in cell wall synthesis. *Proteins*, **48**, 220-226.
13. Gehrke, C.W., Kuo, K.C., McCune, R.A., Gerhardt, K.O. and Agris, P.F. (1982) Quantitative enzymatic hydrolysis of tRNAs: reversed-phase high-performance liquid chromatography of tRNA nucleosides. *J Chromatogr*, **230**, 297-308.

14. Adamiak, R.W., Biala, E., Grzeskowiak, K., Kierzek, R., Kraszewski, A., Markiewicz, W.T., Okupniak, J., Stawinski, J. and Wiewiorowski, M. (1978) The chemical synthesis of the anticodon loop of an eukaryotic initiator tRNA containing the hypermodified nucleoside N6-/N-threonylcarbonyl/-adenosine/t6A/1. *Nucleic Acids Res*, **5**, 1889-1905.

Supplementary Figures and Tables Legends

Supplementary Figures

Fig. S1 Analysis of protein-protein interactions by ITC. A, The titration of YgjD (30 μM) with YeaZ (360 μM). B, The titration of YgjD (20 μM) with $YeaZ^{1-219}$ (200 μM). C, The titration of $YgjD^{S97E}$ (30 μM) with YeaZ (360 μM). D, The titration of YgjD (52 μM) with YjeE (650 μM) in presence of 1.0 mM AMPPNP and $MgCl_2$. E, The titration of YjeE (38 μM) with YeaZ (400 μM) in presence of 1.0 mM ADP and $MgCl_2$. F, The titration of YjeE (20 μM) with YeaZ (240 μM) in presence of 1.0 mM AMPPNP and $MgCl_2$.

Fig. S2 The coordination of ADP and the metal ions in YgjD, YeaZ and YjeE. A, The interfacial ADP is coordinated by resides from both YeaZ (colored in blue) and YgjD (colored in red). B, The coordination of ADP, Fe^{3+} and Mg^{2+} in the active site of YgjD. C, The sequence alignment between *Hi*-YjeE and *Ec*-YjeE superposed by secondary structure elements. D, Comparison of the experimental SAXS curve (black dots) of YjeE with the curve calculated by the program CRYSOL (red line) from the structural model built by $PHYRE^2$. E, The structural superposition of model of *Ec*-YjeE (colored in grey) and the crystal structure of *Hi*-YjeE (colored in cyan, PDB ID: 1HTW). The residues that were subjected for mutational study are highlighted in consistence with C. The dash line in the representation indicates the distance between two atoms. F, Experimental curve (black dots) of the YjeE-dimer superimposed to the calculated curve obtained using the GASBOR program. G, Two monomers of YjeE embedded in a typical envelope produced by GASBOR.

Fig. S3 Analyses of the protein-ligand interaction by ITC. All the titrants and injectants except those in B are supplemented with 500 μM $MgCl_2$. A, Titration of YgjD (30 μM) with AMPCPP (360 μM). B, Titration of the heterodimer YgjD-YeaZ (30 μM) with AMPCPP (360 μM). C, Titration of YjeE (57 μM) with ATPγS (680 μM). D, Titration of YjeE (50 μM) with ADP (600 μM). E, Titration of $YjeE^{Y82A}$ (28 μM) with ATPYS (350 μM). F, Titration of $YjeE^{W109A}$ (41 μM) with ADP (492 μM). G, Titration of YgjD (30 μM) with ADP (360 μM). H, Titration of YeaZ (30 μM) with ADP (360 μM). I, Titration of YeaZ (30 μM) with ATP (360 μM). J, Titration of $YgjD^{E12A}$ (30 μM) with AMPCPP (360 μM). K, Titration of $YjeE^{T43A}$ (33 μM) with ADP (400 μM). L, Titration of $YjeE^{E108A}$ (20 μM) with ADP (260 μM).

Fig. S4 The kinetic characterization of the ATPase activity of the ternary YgjD-YeaZ-YjeE complex. A, Calibration curve for consumption of the NADH by ADP, the calculated slope is -0.002421 μmol^{-1}. B, Kinetics of ATP hydrolysis into ADP by YgjD-YeaZ-2xYjeE as a function of ATP concentration. C, The

correlation between the ATPase activity (black bars) and YjeE monomeric portion (grey bars). The latter was determined by integration of peaks from the Hiload Superdex 75 HR 10/30 size-exclusion chromatogram (not shown). D, The allosteric regulation of the ATPase activity of YjeE by the heterodimer YgjD-YeaZ. The ATPase activity was measured as a function of concentration of YjeE in presence of 2 μM heterodimer YgjD-YeaZ and 4 mM ATP. The data was plotted using allosteric sigmoidal equation (GraphPad).

Fig. S5 ITC analyses of the interaction between the heterodimer YgjD-YeaZ and YjeE. A, The titration of YgjD-YeaZ (23 μM) with YjeE (270 μM) in presence of 1.0 mM AMPPNP and $MgCl_2$. B, The titration of YgjD-YeaZ (32 μM) with YjeE (380 μM) in presence of 1.0 mM AMPPNP and $MgCl_2$. C, The titration of YgjD-YeaZ^{R118A} (40 μM) with YjeE (480 μM) in presence of 1.0 mM AMPPNP and $MgCl_2$. D, The titration of YgjD-YeaZ (20 μM) with YjeE (240 μM). E, The titration of YgjD-YeaZ (20 μM) with YjeE (240 μM) in presence of 1.0 mM ADP and $MgCl_2$. F, The titration of YgjD-YeaZ (20 μM) with YjeE (240 μM) in presence of 1.0 mM ATP and $MgCl_2$. G, The titration of YgjD-YeaZ (23 μM) with YjeE^{E108A} (270 μM) in presence of 1.0 mM AMPPNP and $MgCl_2$. H, The titration of YgjD-YeaZ (30 μM) with YjeE^{T43A} (340 μM) in presence of 1.0 mM AMPPNP and $MgCl_2$. I, The titration of YgjD^{S97E}/YeaZ (30 μM) with YjeE (360 μM) in presence of 1.0 mM AMPPNP and $MgCl_2$.

Fig. S6 The characterization of YgjD-YeaZ protein-protein interaction. A, The determination of oligomeric state in solution of YgjD^{S97R} and YgjD^{F100E} compared to wild type by size exclusion chromatography. B, The interaction of the YeaZ$^{1-219}$ deletion mutant and YgjD by native gel shift. C, Native gel shift analysis of the effect of the mutations on Ser^{97} and Phe^{100} of YgjD on its association with YeaZ and YeaZ/YjeE in presence of 1.0 mM ATP and 2.0 μM $MgCl_2$.

Fig. S7 C18 reverse phase HPLC analyses of the *in vitro* biosynthesis of t^6A. A, Chromatogram of a standard solution containing the bases of C, U, G, t^6A (indicated by asterisk) and A. B, The digested nucleosides of *E. coli* tRNA^{Lys} alone. C, the digested nucleosides of *E. coli* tRNA^{Lys} after enzymatic reaction with YgjD, YeaZ, YjeE and YrdC; the molecular weight of t^6A was determined by MS (represented in the blue square). D, The digested nucleosides of *E. coli* tRNA^{Lys} after enzymatic reaction with YgjD^{E12A}, YeaZ, YjeE and YrdC. E, The digested nucleosides of *E. coli* tRNA^{Lys} after enzymatic reaction with YgjD^{S97E}, YeaZ, YjeE and YrdC. F, The digested nucleosides of *E. coli* tRNA^{Lys} after enzymatic reaction with YgjD, YeaZ^{R118A}, YjeE and YrdC. G, The digested nucleosides of *E. coli* tRNA^{Lys} after enzymatic reaction with YgjD, YeaZ, YjeE^{E108A} and YrdC. H, The digested nucleosides of *E. coli* tRNA^{Lys} after enzymatic reaction with YgjD, YeaZ, YjeE^{W109A} and YrdC.

Fig. S8 SAXS analyses of protein complexes and partners. A, C, E, F, G, SAXS results for YgjD-dimer, YgjD-YeaZ, YgjD-YeaZ$^{1\text{-}219}$-YjeE, YgjD-YeaZ-YjeE^{W109A} and YeaZ-dimer respectively. Comparison of the experimental curve (black dots) with the curve calculated by using CRYSOL (red line) from the model built as described in the text. In all cases the goodness of fit was characterized by the parameter χ given by

$$\chi^2 = \frac{1}{N-1}\sum_j \left[\frac{I_{\exp}(q_j) - cI_{calc}(q_j)}{\sigma(q_j)} \right]^2$$

where N is the number of experimental points, c is a scaling factor, and $I_{calc}(q_j)$ and $s(q_j)$ are the calculated intensity and the experimental error at the scattering vector q_j, respectively. B, SAXS curve for the monomer of YgjD^{S97E}. The blue curve is calculated from the crystal structure of YgjD. The red one is obtained by slightly opening the two domains of the protein. Insert: in blue, the crystal structure; in red, the “open” structure. D, HPLC chromatographic profiles of YgjD-YeaZ alone and in a mixture of large excess of YjeE (4x) in presence of 1.0 mM ADP and 2.0 mM $MgCl_2$. H, The distance distribution functions P(r) for YgjD-YeaZ (blue) and YgjD-YeaZ-YjeE (red) deduced from the scattering curves I(q) using the program GNOM. The two functions come to zero for the same value $r=D_{max}$=90Å.

Supplementary Tables

Table S1 Crystal data and structural refinement statistics.

Table S2 Characteristic parameters of YgjD, YgjD-YeaZ, YjeE and respective complexes calculated from the SAXS experimental curves.

Supplementary Figures

Fig. S1

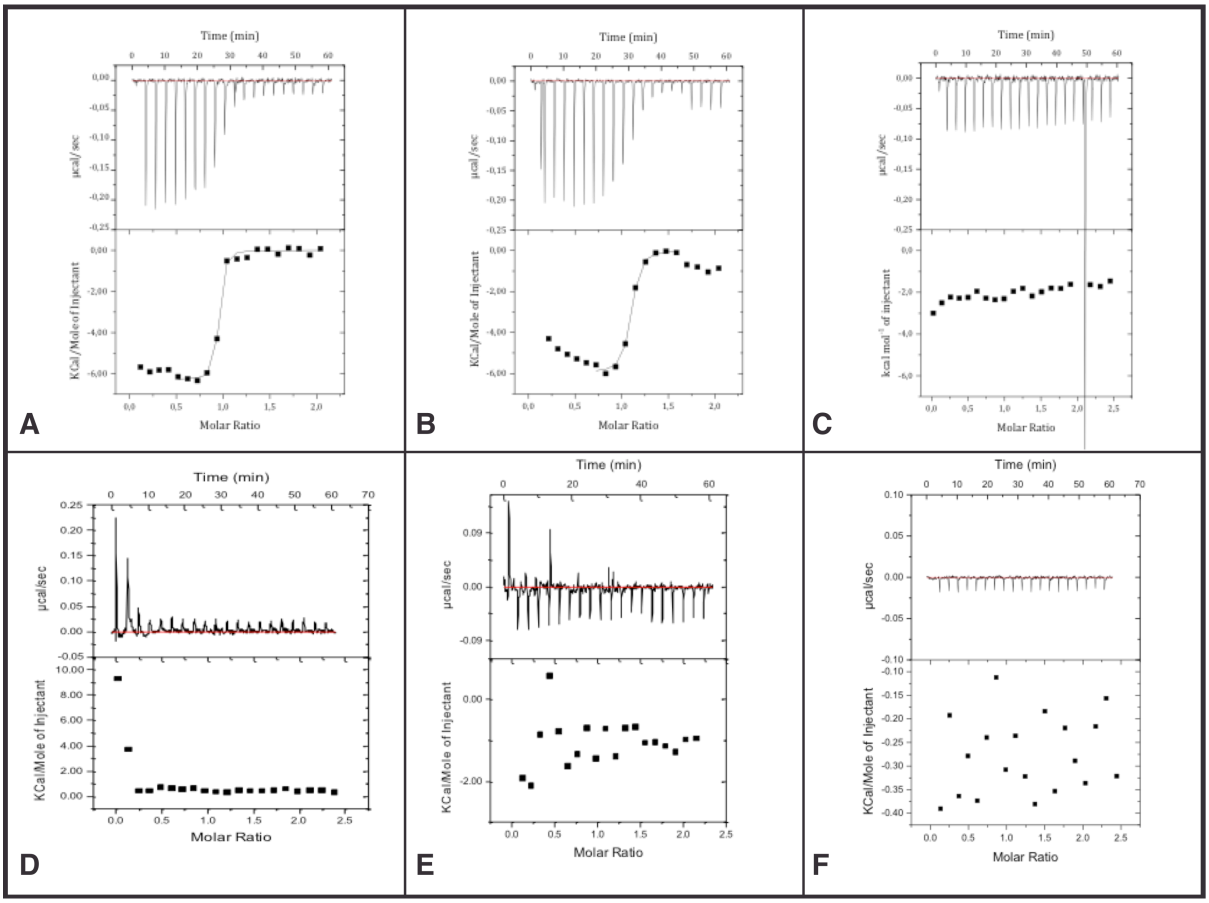

Fig. S2

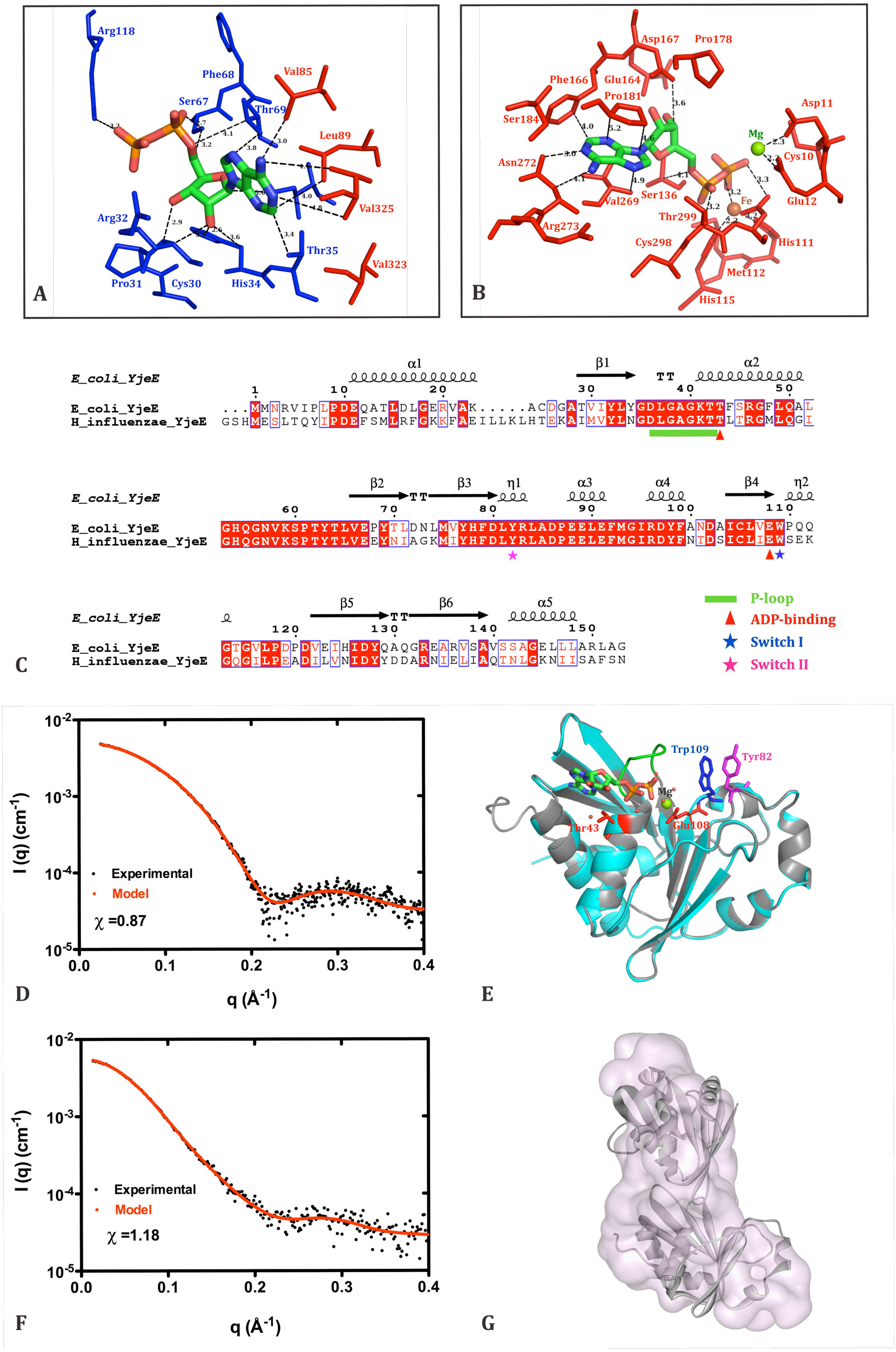

Arg118
Phe68
Ser67
Thr69
Val85
Leu89
Val325
Arg32
Thr35
Val323
Pro31
Cys30
His34
A
Asp167
Pro178
Phe166
Glu164
Pro181
Ser184
Asp11
Mg
Cys10
Asn272
Ser136
Fe
Glu12
Val269
Thr299
Arg273
His111
Cys298
Met112
His115
B
E_coli_YjeE
E_coli_YjeE ...MMNRVIPLPDEQATLDLGERVAK.....ACDGATVIYLYGDLGAGKTTFSRGFLQAL
H_influenzae_YjeE GSHMESLTQYIPDEFSMLRFGKKFAEILLKLHTEKAIMVYLNGDLGAGKTTLTRGMLQGI
E_coli_YjeE
E_coli_YjeE GHQGNVKSPTYTLVEPYTLDNLMVYHFDLYRLADPEELEFMGIRDYFANDAICLVEWPQQ
H_influenzae_YjeE GHQGNVKSPTYTLVEEYNIAGKMIYHFDLYRLADPEELEFMGIRDYFNTDSICLIEWSEK
E_coli_YjeE
E_coli_YjeE GTGVLPDPDVEIHIDYQAQGREARVSAVSSAGELLLARLAG
H_influenzae_YjeE GQGILPEADILVNIDYYDDARNIELIAQTNLGKNIISAFSN
P-loop
ADP-binding
Switch I
Switch II
C
Experimental
Model
χ =0.87
I (q) (cm⁻¹)
q (Å⁻¹)
D
Trp109
Tyr82
Mg
Thr43
Glu108
E
Experimental
Model
χ =1.18
I (q) (cm⁻¹)
q (Å⁻¹)
F
G

Fig. S3

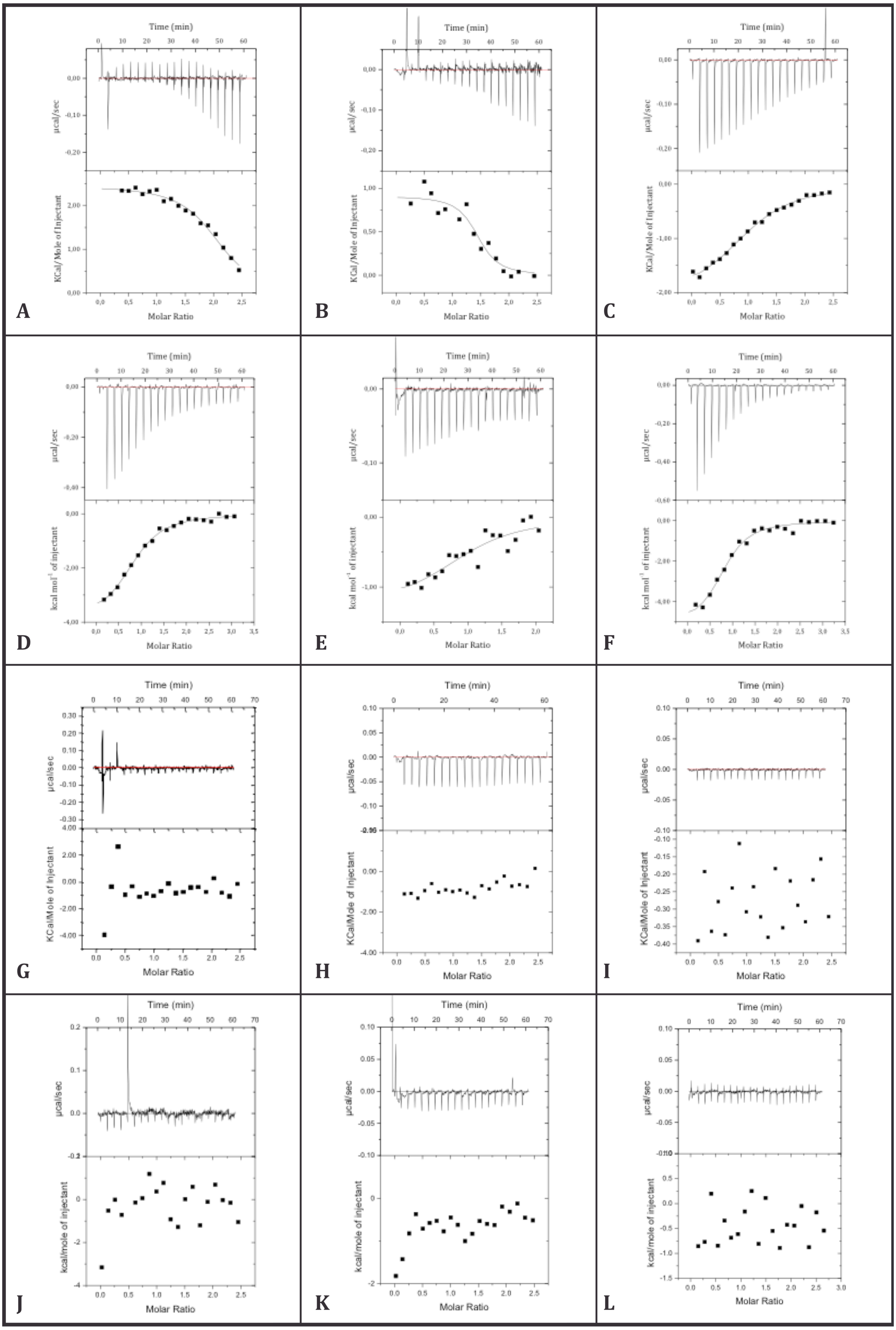

Fig. S4

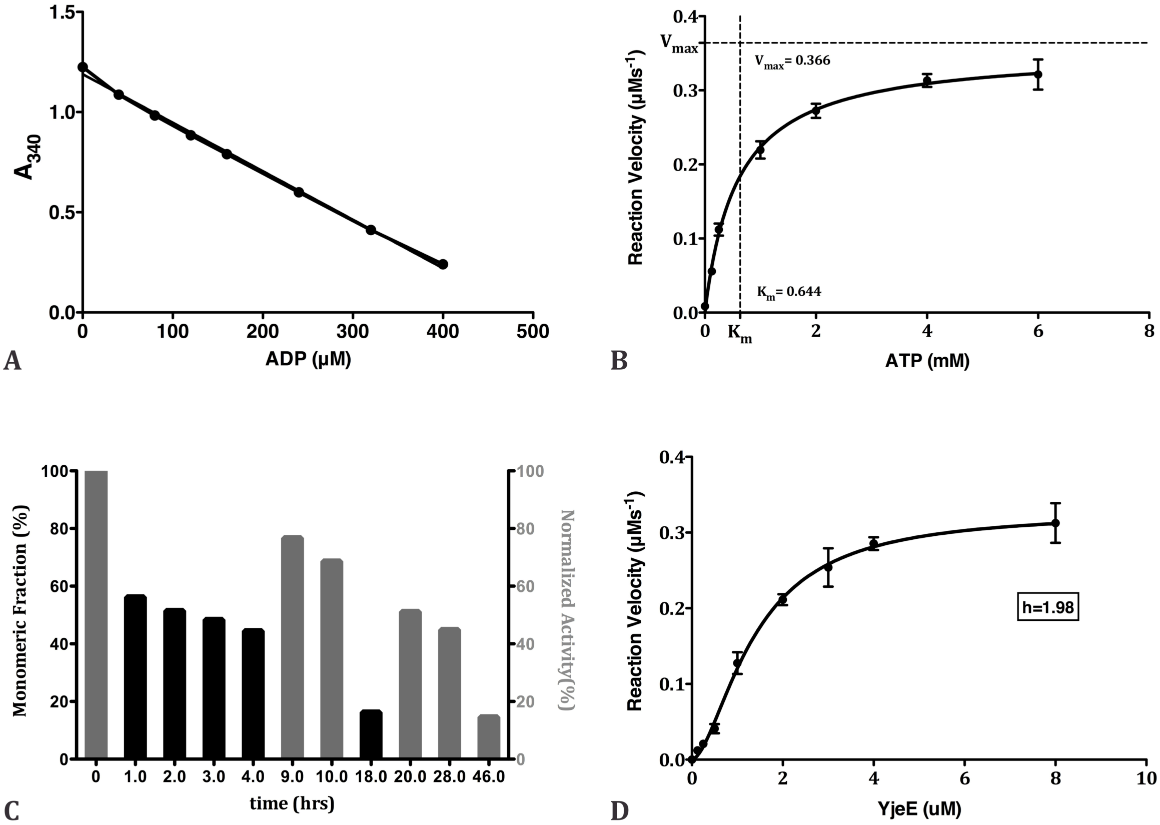
A
A_{340}
ADP (μM)
1.5
1.0
0.5
0.0
0
100
200
300
400
500
B
Reaction Velocity (μMs^{-1})
ATP (mM)
V_{max}
V_{max}= 0.366
K_m= 0.644
K_m
0.4
0.3
0.2
0.1
0.0
0
2
4
6
8
C
Monomeric Fraction (%)
Normalized Activity(%)
time (hrs)
100
80
60
40
20
0
0
1.0
2.0
3.0
4.0
9.0
10.0
18.0
20.0
28.0
46.0
D
Reaction Velocity (μMs^{-1})
YjeE (uM)
h=1.98
0.4
0.3
0.2
0.1
0.0
0
2
4
6
8
10

Fig. S5

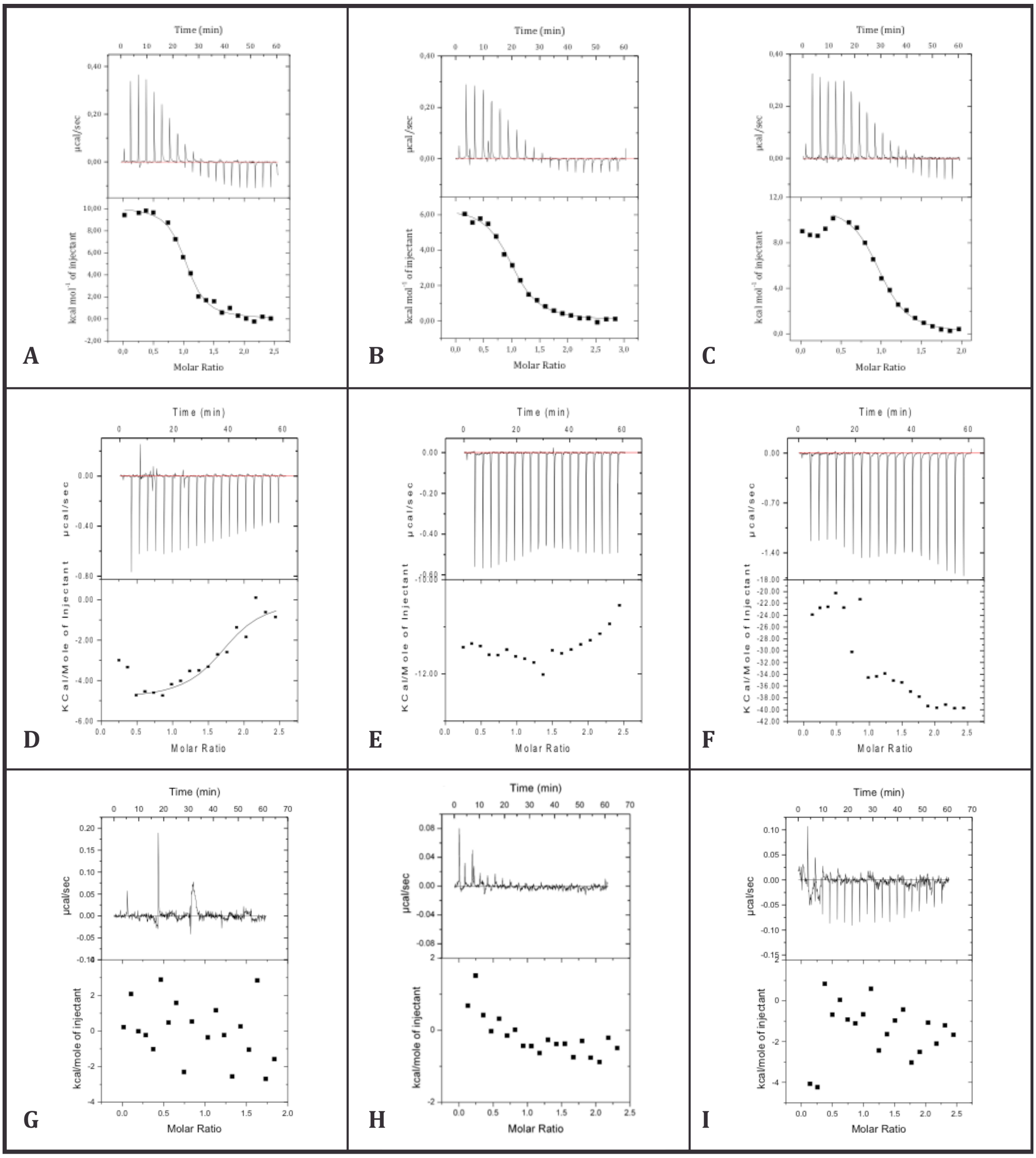

Time (min)
µcal/sec
kcal mol^{-1} of injectant
Molar Ratio
A
B
C
KCal/Mole of Injectant
D
E
F
kcal/mole of injectant
G
H
I

Fig. S6

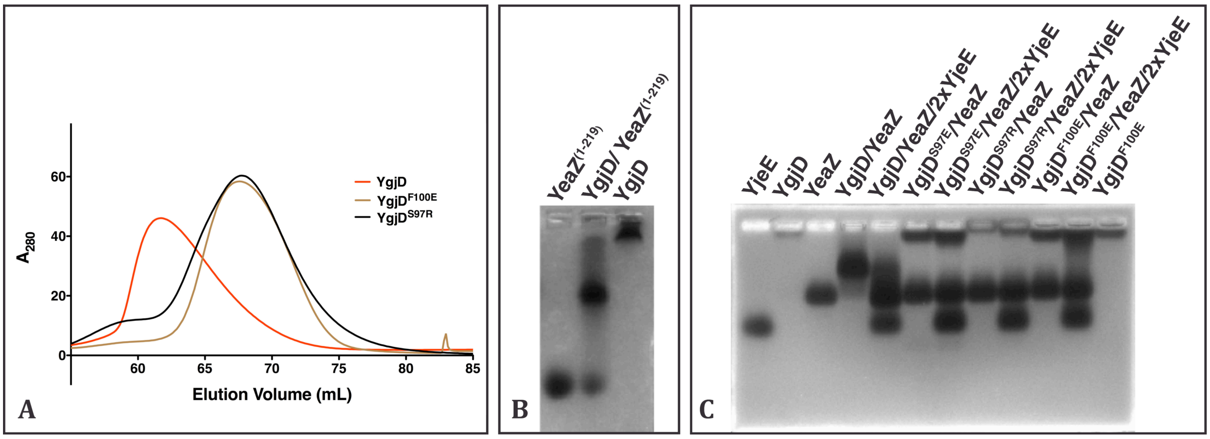

A
A280
60
40
20
0
60
65
70
75
80
85
Elution Volume (mL)
YgjD
YgjDF100E
YgjDS97R
B
YeaZ(1-219)
YgjD/ YeaZ(1-219)
YgjD
C
YjeE
YgjD
YeaZ
YgjD/YeaZ
YgjD/YeaZ/2xYjeE
YgjDS97E/YeaZ
YgjDS97E/YeaZ/2xYjeE
YgjDS97R/YeaZ
YgjDS97R/YeaZ/2xYjeE
YgjDF100E/YeaZ
YgjDF100E/YeaZ/2xYjeE
YgjDF100E

Fig. S7

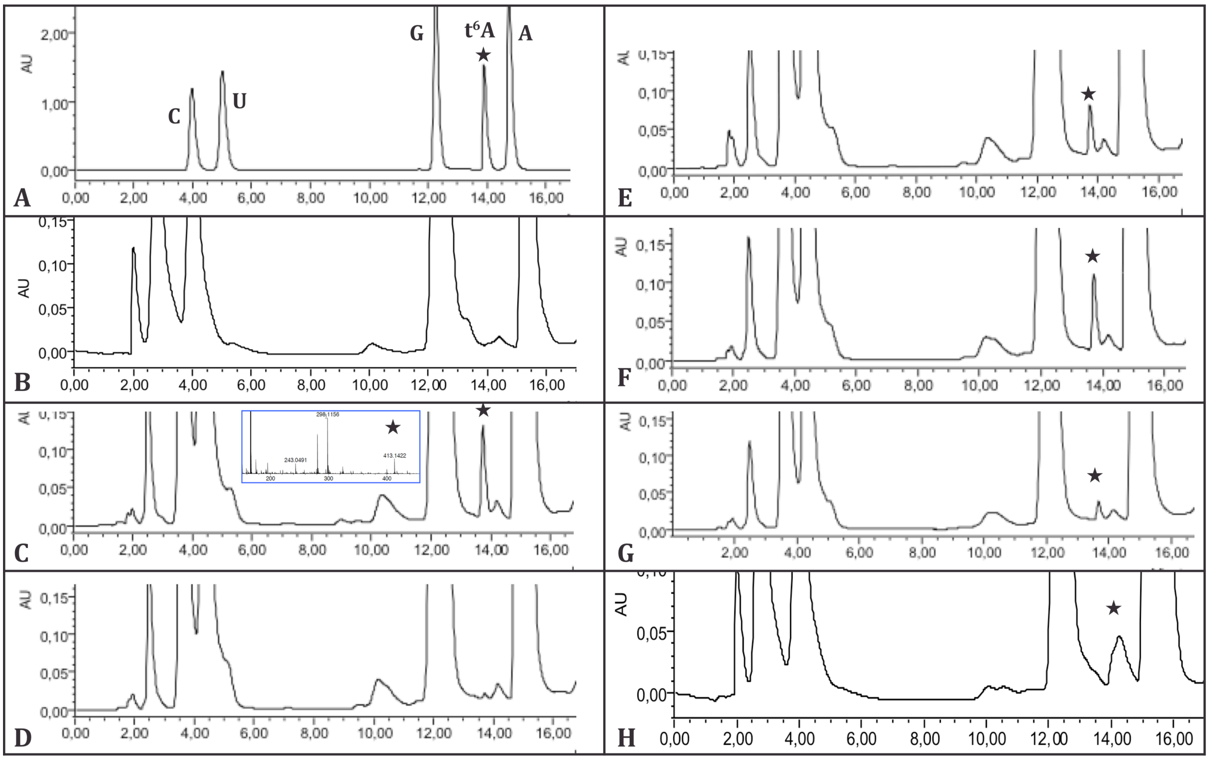
A
B
C
D
E
F
G
H
AU
C
U
G
t6A
A
0,00
2,00
4,00
6,00
8,00
10,00
12,00
14,00
16,00
0,00
0,05
0,10
0,15
1,00
2,00
298,1156
243,0491
413,1422
200
300
400

Fig. S8

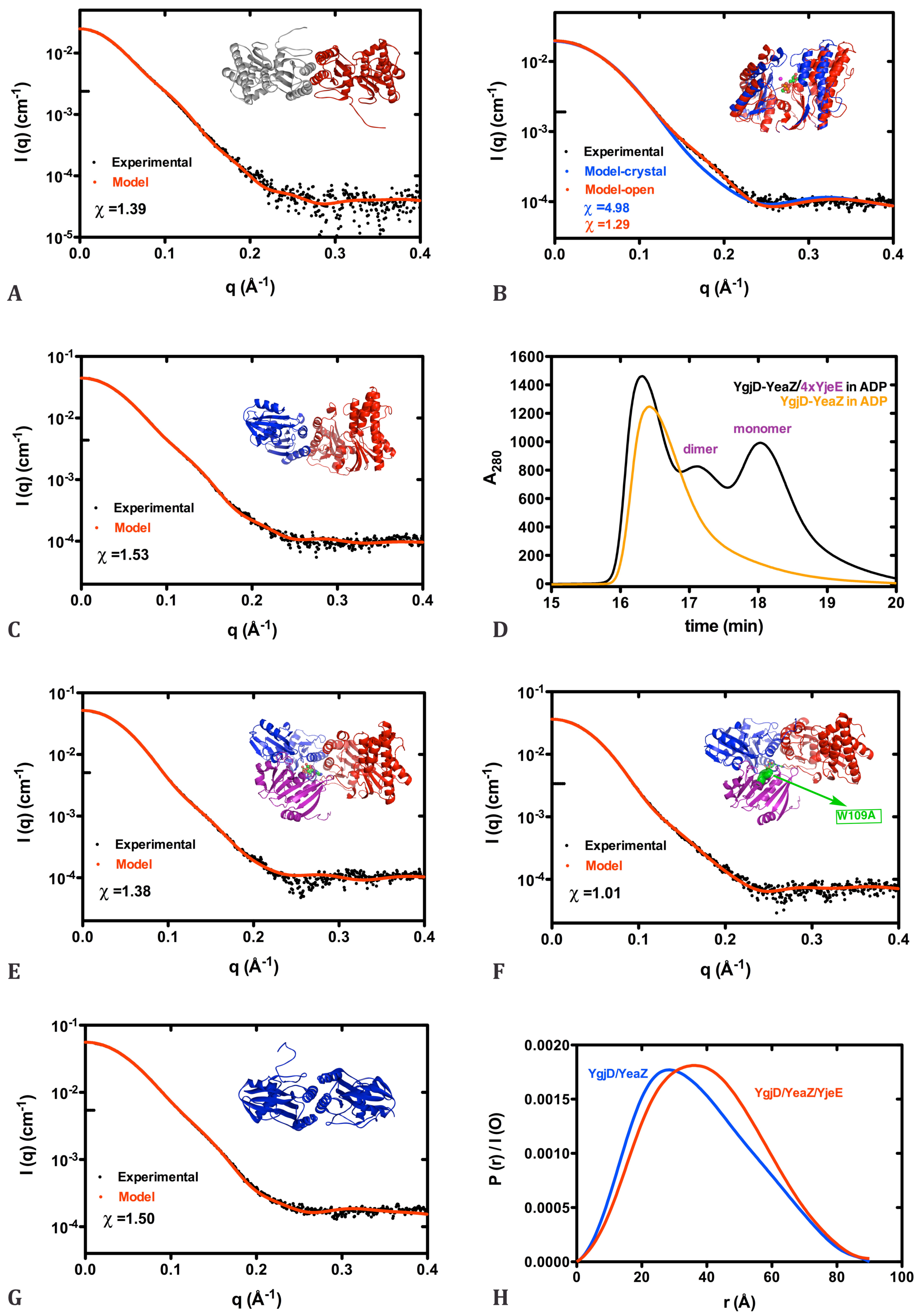

A
I (q) (cm-1)
q (Å-1)
Experimental
Model
χ =1.39
B
Experimental
Model-crystal
Model-open
χ =4.98
χ =1.29
C
Experimental
Model
χ =1.53
D
YgjD-YeaZ/4xYjeE in ADP
YgjD-YeaZ in ADP
dimer
monomer
A280
time (min)
E
Experimental
Model
χ =1.38
F
W109A
Experimental
Model
χ =1.01
G
Experimental
Model
χ =1.50
H
YgjD/YeaZ
YgjD/YeaZ/YjeE
P (r) / I (O)
r (Å)

Supplementary Tables

Table S1 Crystallographic data.

Dataset		YgjD(ADP)/YeaZ(ADP)	$YgjD^{E12A}$(ATP)/YeaZ	$YgjD^{V85E}$(ATP)/YeaZ
**Data Collection**				
Wavelength (Å)		0.98011	0.97857	0.9786
Space group		P1	P1	P1
Cell	a, b, c (Å)	63.62, 68.48, 87.07	63.27 68.07 87.13	63.69 68.08 87.34
	α, β, γ (°)	109.38, 92.66, 117.66	109.3 92.98 117.23	109.31 92.56 117.94
Resolution		49.38-2.25 (2.33-2.25)[a]	49.04-2.33 (2.47-2.33)[a]	42.62-2.23 (2.31-2.23)[a]
R_{sym} (%)		10.4 (57.7)	9.8 (41.5)	8.0 (54.2)
$CC_{1/2}$ (%)		99.5 (74.7)	99.4 (90.8)	92.4 (65.7)
<I/σ(I)>		11.00 (2.46)	11.42 (3.54)	12.21 (2.16)
Complete (%)		97.93 (95.69)	96.9 (92.5)	99.7 (84.0)
Multiplicity		3.97	3.18	3.25
**Refinement statistics**				
Resolution		49.38 - 2.25	49.04 - 2.33	42.62 - 2.23
Unique Refl.		55653(5436)	49021(7525)	53417(3359)
R_{work}/R_{free} (%)		20.8/25.5	20.3/25.4	18.6/23.2
Total Atoms		9012	8711	8372
B-factors		34.4	32.8	40.7
RMS(bonds)		0.011	0.012	0.011
RMS(angles)		1.41	1.37	1.31
**Ramachandran Plot**				
Favored (%)		92.2	91.7	91.6
Allowed (%)		7.5	7.9	8.0
Disallowed (%)		0.2	0.4	0.3
PDB code		4WOS	4WQ4	4WQ5

[a] Values in parentheses are for the highest-resolution shell.
Rsym=$\sum_h \sum_i |I_{hi} - <Ih>| / \sum_h \sum_i I_{hi}$, were I_{hi} is the *i*th observation of the reflection h, while <Ih> is the mean intensity of reflection h.
Rfactor =$\sum_i [|F_o| - |F_c|]/|F_o|$. Rfree was calculated with a small fraction (5%) of randomly selected reflections.

Table S2. Structural parameters extracted from SAXS data.

	Quaternary state	R_g Guinier (Å)	R_g P(r) (Å)	D_{max} (Å)	MW (kDa)[a]	Theoretical MW(kDa)[b]	χ
**YgjD-WT**	dimer	32.0	32.9	110	74.0	73.7	1.31
**YgjD^{S97E}**	monomer	23.3	23.3	74	37.9	36.8	1.29
**YgjD-YeaZ**	monomer	29.0	29.2	90	66.5	62.8	1.53
**YgjD-YeaZ-YjeE**	monomer	29.3	29.0	90	81.3	80.7	1.42
**YgjD-YeaZ$^{1\text{-}219}$-YjeE**	monomer	28.9	28.8	89	75.9	78.5	1.38
**YgjD-YeaZ-YjeE^{W109A}**	monomer	29.0	28.9	90	79.2	80.7	1.01
**YjeE**	monomer	16.6	16.5	54	17.1	17.9	0.86
**YjeE**	dimer	24.3	24.3	76	38.2	35.8	1.18
**YeaZ**	dimer	26.3	26.4	84	51.4	52.0	1.50

[a], Molar massM obtained from the whole I(q) curve (q_{max}=0.3Å) using the macromolecule volume and the SAXS-MoW method available at http://www.if.sc.usp.br/~saxs/.

[b], The theoretical masses were derived from the sequence taking into account the tags in each case.
